# Development and Validation of an 18-Gene Urine Test for High-Grade Prostate Cancer

**DOI:** 10.1001/jamaoncol.2024.0455

**Published:** 2024-04-18

**Authors:** Jeffrey J. Tosoian, Yuping Zhang, Lanbo Xiao, Cassie Xie, Nathan L. Samora, Yashar S. Niknafs, Zoey Chopra, Javed Siddiqui, Heng Zheng, Grace Herron, Neil Vaishampayan, Hunter S. Robinson, Kumaran Arivoli, Bruce J. Trock, Ashley E. Ross, Todd M. Morgan, Ganesh S. Palapattu, Simpa S. Salami, Lakshmi P. Kunju, Scott A. Tomlins, Lori J. Sokoll, Daniel W. Chan, Sudhir Srivastava, Ziding Feng, Martin G. Sanda, Yingye Zheng, John T. Wei, Arul M. Chinnaiyan

**Affiliations:** 1 Department of Urology, Vanderbilt University Medical Center, Nashville, Tennessee; 2 Vanderbilt-Ingram Cancer Center, Nashville, Tennessee; 3 Department of Pathology, University of Michigan, Ann Arbor; 4 Department of Biostatistics, Fred Hutchinson Cancer Research Center, Seattle, Washington; 5 Departments of Pathology and Urology, Johns Hopkins University School of Medicine, Baltimore, Maryland; 6 Department of Urology, Northwestern University Feinberg School of Medicine, Chicago, Illinois; 7 Department of Urology, University of Michigan, Ann Arbor; 8 Strata Oncology, Ann Arbor, Michigan; 9 Division of Cancer Prevention, National Institutes of Health, Bethesda, Maryland; 10 Department of Urology, Emory University, Atlanta, Georgia; 11 Howard Hughes Medical Institute, Chevy Chase, Maryland

## Abstract

**Importance:**

Benefits of prostate cancer (PCa) screening with prostate-specific antigen (PSA) alone are largely offset by excess negative biopsies and overdetection of indolent cancers resulting from the poor specificity of PSA for high-grade PCa (ie, grade group [GG] 2 or greater).

**Objective:**

To develop a multiplex urinary panel for high-grade PCa and validate its external performance relative to current guideline-endorsed biomarkers.

**Design, Setting, and Participants:**

RNA sequencing analysis of 58 724 genes identified 54 markers of PCa, including 17 markers uniquely overexpressed by high-grade cancers. Gene expression and clinical factors were modeled in a new urinary test for high-grade PCa (MyProstateScore 2.0 [MPS2]). Optimal models were developed in parallel without prostate volume (MPS2) and with prostate volume (MPS2+). The locked models underwent blinded external validation in a prospective National Cancer Institute trial cohort. Data were collected from January 2008 to December 2020, and data were analyzed from November 2022 to November 2023.

**Exposure:**

Protocolized blood and urine collection and transrectal ultrasound-guided systematic prostate biopsy.

**Main Outcomes and Measures:**

Multiple biomarker tests were assessed in the validation cohort, including serum PSA alone, the Prostate Cancer Prevention Trial risk calculator, and the Prostate Health Index (PHI) as well as derived multiplex 2-gene and 3-gene models, the original 2-gene MPS test, and the 18-gene MPS2 models. Under a testing approach with 95% sensitivity for PCa of GG 2 or greater, measures of diagnostic accuracy and clinical consequences of testing were calculated. Cancers of GG 3 or greater were assessed secondarily.

**Results:**

Of 761 men included in the development cohort, the median (IQR) age was 63 (58-68) years, and the median (IQR) PSA level was 5.6 (4.6-7.2) ng/mL; of 743 men included in the validation cohort, the median (IQR) age was 62 (57-68) years, and the median (IQR) PSA level was 5.6 (4.1-8.0) ng/mL. In the validation cohort, 151 (20.3%) had high-grade PCa on biopsy. Area under the receiver operating characteristic curve values were 0.60 using PSA alone, 0.66 using the risk calculator, 0.77 using PHI, 0.76 using the derived multiplex 2-gene model, 0.72 using the derived multiplex 3-gene model, and 0.74 using the original MPS model compared with 0.81 using the MPS2 model and 0.82 using the MPS2+ model. At 95% sensitivity, the MPS2 model would have reduced unnecessary biopsies performed in the initial biopsy population (range for other tests, 15% to 30%; range for MPS2, 35% to 42%) and repeat biopsy population (range for other tests, 9% to 21%; range for MPS2, 46% to 51%). Across pertinent subgroups, the MPS2 models had negative predictive values of 95% to 99% for cancers of GG 2 or greater and of 99% for cancers of GG 3 or greater.

**Conclusions and Relevance:**

In this study, a new 18-gene PCa test had higher diagnostic accuracy for high-grade PCa relative to existing biomarker tests. Clinically, use of this test would have meaningfully reduced unnecessary biopsies performed while maintaining highly sensitive detection of high-grade cancers. These data support use of this new PCa biomarker test in patients with elevated PSA levels to reduce the potential harms of PCa screening while preserving its long-term benefits.

## Key Points

**Question:**

Can a new 18-gene urinary test for high-grade prostate cancer (ie, grade group [GG] 2 or greater) improve prostate-specific antigen (PSA) screening outcomes relative to existing biomarker tests?

**Findings:**

In this diagnostic study including 761 men in the development cohort and 743 men in the validation cohort, novel cancer-specific and high-grade cancer-specific genes were identified from RNA sequencing data and optimally modeled in a development cohort, yielding an 18-gene test for high-grade prostate cancer. Applying a testing approach with 95% sensitivity for high-grade prostate cancer to an external validation population, use of the 18-gene test would have reduced the number of unnecessary biopsies performed relative to current guideline-endorsed tests.

**Meaning:**

The new 18-gene prostate cancer test may reduce more burdensome additional testing (eg, imaging and biopsy) while maintaining highly sensitive detection of high-grade cancer in patients undergoing PSA screening.

## Teaser

This report describes the development of an 18-gene urine panel for high-grade prostate cancer and validates its external performance relative to current guideline-endorsed biomarkers.

## Introduction

Prostate cancer (PCa) remains the most commonly diagnosed malignancy and a leading cause of cancer death worldwide.^1^ The European Randomized Study of Screening for PCa and Göteborg Randomized Prostate Cancer Screening trial showed significant reductions in cancer mortality for men participating in prostate-specific antigen (PSA)–based screening.^2,3^ At the same time, these studies confirmed that PSA screening leads to unnecessary invasive biopsies in men without cancer and frequent overdiagnosis of low-grade, indolent cancers (grade group [GG] 1).^4^ In response to this, current clinical guidelines offer that men with an elevated PSA level undergo multiparametric magnetic resonance imaging (mpMRI), if available, or biomarker testing for risk stratification prior to biopsy.^5,6^

Indeed, use of prostate mpMRI with targeted biopsy has improved detection of clinically significant, high-grade cancer (ie, cancer of GG 2 or greater) in men with tumors visible on mpMRI.^7^ While these data support prebiopsy mpMRI in patients requiring biopsy, the use of negative findings on mpMRI to rule out high-grade cancers in men with elevated PSA levels is not well supported. Population-level data spanning academic and community settings reveal a negative predictive value (NPV) of only 77% for high-grade cancers,^8^ and subjective interpretation of mpMRI is highly problematic, with NPVs as low as 63% by site and 40% among radiologists.^9,10^ Thus, even following negative findings on mpMRI, its limited sensitivity merits biopsy in a substantial proportion of men. Moreover, there are practical reasons mpMRI may not be feasible for populationwide use after PSA, including its resource burden and limited availability in the community setting.^11,12^

Objective, noninvasive biomarker tests could be a more practical option. Current National Comprehensive Cancer Network (NCCN) guidelines offer 6 blood-based and urine-based biomarker tests, each including 3 or fewer markers of PCa (ie, cancer of any grade).^5^ While consistently outperforming PSA alone,^13^ these assays have not evolved to reflect current understanding of PCa biology. For one, given the minimal metastatic potential of low-grade cancers, contemporary practice is focused on detecting high-grade cancers, while reducing overdiagnosis of low-grade disease.^5^ Thus, assays based solely on markers associated with cancer of any grade have limited biologic specificity for high-grade cancers. Moreover, assays including only 2 to 3 biomarkers simply cannot capture the multitude of diverse molecular pathways driving lethal disease.^14,15^

We hypothesized that augmenting the prior generation of cancer-associated biomarkers with novel molecules selectively expressed by high-grade, aggressive cancers would improve testing accuracy. Leveraging multi-institutional transcriptomic data,^14,16,17^ we identified novel genes specifically overexpressed by high-grade cancers. We then adopted multiplex polymerase chain reaction (PCR)–based technology to evaluate 54 candidate markers in a development cohort, deriving an optimal 18-gene assay for standard clinical use. Finally, we performed blinded external validation of the new assay, including direct comparison with currently endorsed biomarker tests.

## Methods

Institutional review board approval was obtained from the University of Michigan Institutional Review Board and at each site, and all participants provided written informed consent. This study followed the Standards for Reporting of Diagnostic Accuracy (STARD) reporting guideline.^18^

### Biomarker Discovery

**Figure 1.**
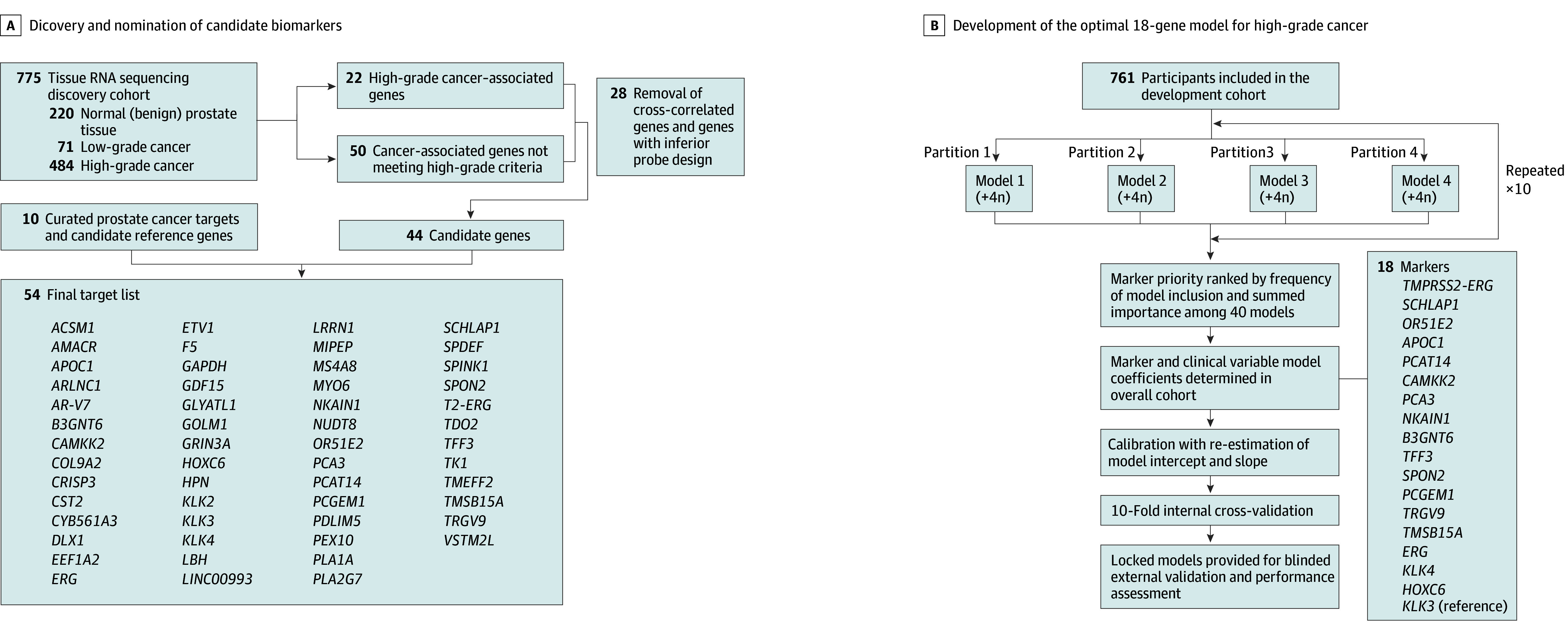
Biomarker Discovery and Development of the MyProstateScore 2.0 Urinary Test for High-Grade Prostate Cancer A, Discovery and nomination of candidate biomarkers for the multiplex urinary panel. Biomarker discovery was performed using RNA sequencing data from 220 benign prostates, 71 with cancers of grade group 1, and 484 with cancers of grade group 2 or greater available through the Cancer Genome Atlas, the Genotype-Tissue Expression portal, and the University of Michigan. A total of 72 markers met predefined criteria. Of these, quantitative polymerase chain reaction probes could not be successfully designed for 19, and 9 genes were highly cross-correlated, resulting in exclusion from the candidate panel. The remaining 44 transcripts meeting nomination criteria were supplemented with 10 curated genes to yield a 54-gene candidate panel. B, Development of the optimal 18-gene model for high-grade cancer. To avoid multicollinearity in regression models, highly correlated variables were identified and removed with a stepwise procedure. We assessed 3 model-building approaches: (1) logistic regression with stepwise feature selection, (2) logistic regression with recursive feature elimination, and (3) regularized logistic regression with elastic net. Performance of each model-building approach was quantified as the area under the receiver operating characteristic curve on repeated cross-validation (10-fold cross-validation repeated 3 times) with upsampling of the minor class to yield balanced classes. Elastic net modeling yielded the highest median area under the curve and was used for development. Using an ensemble approach, the development set was randomly divided into 4 partitions, and the model yielding the highest area under the curve was identified for each partition. This approach was repeated 10 times with different random seeds, yielding 40 elastic net models in total. For each candidate gene, the frequency of model inclusion and importance to high-grade prostate cancer detection was tabulated across models. Based on analysis of optimal feature size and technical features of the OpenArray platform (Thermo Fisher Scientific), the 17 biomarkers providing optimal discriminative accuracy for prostate cancer of grade group 2 or greater were included with standard clinical variables and the normalization gene *KLK3* in the MyProstateScore 2.0 model (without prostate volume) and MyProstateScore 2.0 Plus model (with prostate volume). Models were calibrated and internally cross-validated prior to external validation.

The original MyProstateScore (MPS) test incorporates prostate cancer antigen 3 (*PCA3*) and *TMPRSS2:ERG* gene fusion expression with serum PSA level to estimate risk of high-grade cancers and is endorsed by NCCN guidelines for prebiopsy risk stratification.^5,19^ To derive a gene panel for high-grade cancers, we performed differential expression analysis of 58 724 genetic targets in multi-institutional RNA sequencing data (Figure 1; eFigures 1 and 2 in Supplement 1 and the eTable in Supplement 2). A total of 72 genes met predefined nomination criteria for cancer (n = 50) or high-grade cancer (n = 22) (eTable 1 in Supplement 1). Removal of collinear genes and those without PCR primers resulted in 44 candidate markers (eFigures 1 to 3 in Supplement 1). These were supplemented with 10 previously described PCa-associated or reference genes, yielding a 54-gene candidate panel.

### Model Development

#### Development Cohort

Prebiopsy urine has been prospectively collected at the University of Michigan Prostate Specialized Program of Research Excellence under a National Cancer Institute (NCI) Early Detection Research Network (EDRN) protocol approved by the University of Michigan Institutional Review Board since 2008. First-catch urine was obtained following digital rectal examination and was mixed with RNA stabilization buffer and frozen at −70 °C.^20^ The development cohort included patients presenting for 12-core or greater prostate biopsy due to elevated PSA levels (3-10 ng/mL; to convert to micrograms per liter, multiply by 1) from 2008 to 2020. In accordance with guidelines,^5^ we excluded patients with PCa. Based on proposed use of this test as a pre-mpMRI, prebiopsy test to rule out the need for mpMRI or biopsy, we excluded men with a history of prostate mpMRI and targeted biopsy.

#### Multiplex Quantitative PCR OpenArray Profiling

OpenArray technology (Thermo Fisher Scientific) is a high-throughput real-time quantitative PCR (qPCR) method for rapid screening of multiple TaqMan assays. RNA isolation, extraction, and complementary DNA synthesis were performed (eFigure 4 in Supplement 1).

#### Model Building and Calibration

We assessed the 54-gene candidate panel using multiple model-building strategies (Figure 1). Clinical factors consistently associated with PCa (age, race, digital rectal examination findings, PSA level, family history of prostate cancer, and prior negative biopsy)^21^ were locked into models a priori. Because prostate volume improves predictive value^22,23^ but is not available for all patients, we developed a second model including volume for use when volume is clinically available (ie, previous biopsy or mpMRI). Test outputs were standardized to represent the percentage likelihood of detecting high-grade cancers (0% to 100%). The optimal 18-gene model without prostate volume (MPS2) and with prostate volume (MPS2+) were calibrated (eFigure 5 in Supplement 1) to account for differences in outcome prevalence between cohorts,^21,24,25^ and the calibrated models were locked for external validation. The MPS2 test is owned by LynxDx, which has obtained an exclusive license for commercialization from the University of Michigan.

### Model Validation

#### External Validation Cohort

The validation cohort consisted of patients in the prospective NCI EDRN PCA3 Evaluation Trial. This trial enrolled consecutive patients presenting for biopsy across 11 academic centers, primarily due to elevated PSA levels or abnormal digital rectal examination findings (eTable 2 in Supplement 1).^20^ Patient race was self-reported via a questionnaire; selectable options included American Indian or Alaska Native, Asian, Black, Native Hawaiian or Other Pacific Islander, White, other, or unknown race. Black race was considered pertinent to this study based on the known association of Black race with PCa incidence, outcomes, and molecular subtypes.^26^ Because such associations are not well established for other racial groups and because racial groups other than Black and White are frequently misclassified,^27^ racial groups were categorized as Black or other race.

#### Specimens and Laboratory Analysis

Deidentified urine specimens were shipped to the University of Michigan for OpenArray profiling. Laboratory procedures were conducted per the identical protocol used in development. We derived a multiplex 2-gene model (*HOXC6* and* DLX1)* and a multiplex 3-gene model (*PCA3*, *ERG*, and* SPDEF)*. These genes are measured in the commercially available SelectMDx and ExoDx Prostate Intelliscore (EPI) tests, respectively. The multiplex models considered herein were independently derived based on gene expression measured using the OpenArray qPCR platform and are not proposed to represent the commercial products. Serum PSA, free PSA, and [−2]proPSA were measured using the Access 2 Immunoassay System (Beckman Coulter) at the Johns Hopkins EDRN Laboratory.

#### Blinded Validation and Comparative Analysis

Expression data and model coefficients were available to 2 investigators (C.X. and Y. Zheng) at the NCI EDRN for predefined validation. Locked model coefficients from development were used to generate outputs of the derived multiplex 2-gene model, derived multiplex 3-gene model, MPS2, and MPS2+. The original MPS was calculated using qPCR-based *PCA3* and *TMPRSS2:ERG* scores^19^; a subset of data were previously described.^28^ Prostate Health Index (PHI) was calculated using the formula ([−2]proPSA/free PSA) × √(PSA).

Comparative analysis included PSA, the Prostate Cancer Prevention Trial risk calculator,^21^ PHI, the derived multiplex 2-gene and 3-gene models, MPS,^19^ MPS2, and MPS2+. The primary outcome was cancer of GG 2 or greater on biopsy; cancer of GG 3 or greater was secondarily assessed. Diagnostic potential was visualized by receiver operating characteristic (ROC) curves and quantified by area under the ROC curve (AUC) using R package pROC.^29^ For the development cohort, mean AUC from repeated 10-fold cross-validation was reported. For the validation cohort, 95% CIs of AUCs were computed with 2000 stratified bootstrap.

We sought to illustrate test performance using a straightforward, clinically applicable approach. As described,^30^ considering prevalence of high-grade cancers in testing populations (17% to 31%),^31,32,33,34,35^ relative harms of false-negative and false-positive testing results,^36^ and the proposed role of biomarkers for rule-out testing,^37,38^ we assessed thresholds providing 95% sensitivity for high-grade cancer. Performance measures were calculated using the confusionMatrix function of R package caret. Given disparate risk profiles of initial and repeat biopsy populations,^39,40,41^ analyses were carried out in each subpopulation.

Decision curve analysis (DCA) was used to quantify net benefit of each biomarker on the decision to undergo biopsy compared with (1) biopsying all patients and (2) biopsying no patients.^42^ Considering a more than 20% risk of high-grade cancer justifies performing biopsy and a less than 5% risk justifies foregoing biopsy in most patients,^43^ we assessed threshold probabilities spanning this clinically relevant range. DCA was performed using dca in the R package dcurves.

### Statistical Analysis

Statistical analyses were performed using R version 4.1.1 (The R Foundation). Two-tailed tests were used for all comparisons, and *P* values less than .05 were considered statistically significant.

## Results

### Model Development

Among 815 participants in the development cohort, qPCR yielded valid results in 761 (93.4%) (eFigure 4 in Supplement 1). The median (IQR) age was 63 (58-68) years, and the median (IQR) PSA level was 5.6 (4.6-7.2) ng/mL (Table 1). On study biopsy, 293 men (38.5%) had cancer of GG 2 or greater. The contribution of candidate genes to model predictions was quantified across elastic net models (Figure 1; eTable 3 in Supplement 1). The final MPS2 model included clinical variables and the 17 most informative markers, including 13 from the discovery analysis (4 high-grade cancer–specific genes [*APOC1*, *B3GNT6*, *NKAIN1*, and *SCHLAP1*] and 9 cancer-specific genes [*PCGEM1*, *SPON2*,* TRGV9*,* PCA3*,* OR51E2*,* CAMKK2*,* TFF3*,* PCAT14*, and* TMSB15A*]), 4 curated markers (*HOXC6*, *ERG*, *TMPRSS2:ERG*, and *KLK4*), plus the reference gene *KLK3* (eTable in Supplement 3). Model coefficients were determined in the overall cohort (eTable 4 in Supplement 1). Calibration and internal cross-validation were performed (eFigures 5 and 6 in Supplement 1), and the MPS2 models were locked for external validation.

**Table 1.** Characteristics of the Development and Validation Populations Overall and Stratified by Pathologic Findings on Prostate Biopsy

Characteristic	Median (IQR)									
	Development cohort					External validation cohort				
	Total (n = 761)	Negative (n = 362)	GG 1 (n = 106)	Negative or GG 1 (n = 468)	GG ≥2 (n = 293)	Total (n = 743)	Negative (n = 452)	GG 1 (n = 140)	Negative or GG 1 (n = 592)	GG ≥2 (n = 151)
Age, y	63 (58-68)	62 (57-67)	64 (57-68)	63 (57-68)	64 (58-69)	62 (57-68)	62 (57-67)	63 (57-67)	62 (57-68)	64 (59-70)
Race, No. (%)^a^										
Black	33 (4)	12 (3)	4 (4)	16 (3)	17 (6)	95 (13)	51 (11)	19 (14)	70 (12)	25 (17)
Other race	728 (96)	350 (97)	102 (96)	452 (97)	276 (94)	648 (87)	401 (89)	121 (86)	522 (88)	126 (83)
Positive family history, No. (%)	206 (27)	88 (24)	28 (26)	116 (25)	90 (31)	212 (29)	118 (26)	46 (33)	164 (28)	48 (32)
Previous negative biopsy, No. (%)	163 (21)	105 (29)	22 (21)	127 (27)	36 (12)	247 (33)	196 (43)	33 (24)	229 (39)	18 (12)
Abnormal DRE, No. (%)	104 (14)	34 (9)	4 (4)	38 (8)	66 (23)	139 (19)	72 (16)	16 (11)	88 (15)	51 (34)
Prostate volume, mL^b^	48 (36-66)	56 (42-76)	47 (37-61)	53 (40-72)	41 (30-54)	43 (32-60)	48 (35-69)	40 (29-52)	46 (34-65)	36 (28-47)
PSA, ng/mL	5.6 (4.6-7.2)	5.6 (4.6-6.8)	5.55 (4.6-7.0)	5.6 (4.6-6.9)	5.6 (4.7-7.5)	5.6 (4.1-8.0)	5.5 (4.0-8.0)	5.3 (4.3-7.0)	5.4 (4.0-7.7)	6.2 (4.7-8.9)
PSA density, ng/mL^2^^c^	0.12 (0.08-0.16)	0.10 (0.07-0.14)	0.12 (0.09-0.16)	0.10 (0.07-0.14)	0.15 (0.10-0.20)	0.12 (0.08-0.19)	0.11 (0.07-0.17)	0.12 (0.09-0.18)	0.11 (0.08-0.17)	0.17 (0.12-0.31)
PHI	NA	NA	NA	NA	NA	40.5 (30.0-55.0)	36.5 (27.7-47.6)	40.8 (32.2-50.7)	37.4 (28.4-49.5)	57.5 (44.9-86.8)
Derived multiplex 2-gene model	NA	NA	NA	NA	NA	0.46 (0.33-0.67)	0.40 (0.28-0.59)	0.45 (0.36-0.64)	0.42 (0.31-0.60)	0.70 (0.49-0.90)
Derived multiplex 3-gene model	NA	NA	NA	NA	NA	0.45 (0.31-0.60)	0.38 (0.28-0.51)	0.53 (0.39-0.66)	0.41 (0.29-0.55)	0.59 (0.46-0.70)
MPS	37 (20-58)	26 (14-42)	42 (24-63)	29 (16-48)	51 (33-72)	35 (17-56)	26 (12-44)	42 (26-65)	30 (15-49)	55 (37-72)
MPS2^d^	0.16 (0.05-0.39)	0.07 (0.03-0.16)	0.15 (0.06-0.30)	0.07 (0.03-0.19)	0.40 (0.20-0.61)	0.13 (0.05-0.37)	0.08 (0.03-0.19)	0.20 (0.08-0.43)	0.10 (0.04-0.24)	0.44 (0.23-0.69)
MPS2+^d^	0.14 (0.05-0.42)	0.06 (0.02-0.14)	0.14 (0.06-0.32)	0.07 (0.03-0.17)	0.44 (0.22-0.68)	0.15 (0.05-0.43)	0.08 (0.03-0.21)	0.25 (0.09-0.48)	0.11 (0.04-0.30)	0.54 (0.27-0.79)

Abbreviations: DRE, digital rectal examination; GG, grade group; MPS, MyProstateScore; MPS2, MyProstateScore 2.0; MPS2+, MyProstateScore 2.0 plus prostate volume; NA, not applicable; PHI, Prostate Health Index; PSA, prostate-specific antigen.

SI conversion factor: To convert PSA to μg/L, multiply by 1.

^a^ Race was self-reported by participants via a questionnaire. Black race was pertinent to the current study due to the well-established association of race with prostate cancer incidence, outcomes, and tumor molecular subtypes.^26^ The other race category includes American Indian or Alaska Native, Asian, Native Hawaiian or Other Pacific Islander, White, other, and unknown race.

^b^ Measured by transrectal ultrasound.

^c^ PSA density equals serum PSA divided by prostate volume.

^d^ MPS2 and MPS2+ values are reported on a continuous scale as the likelihood of cancer of GG 2 or greater detection on biopsy.

### External Validation and Comparative Analysis

#### Overall Study Population

**Figure 2.**
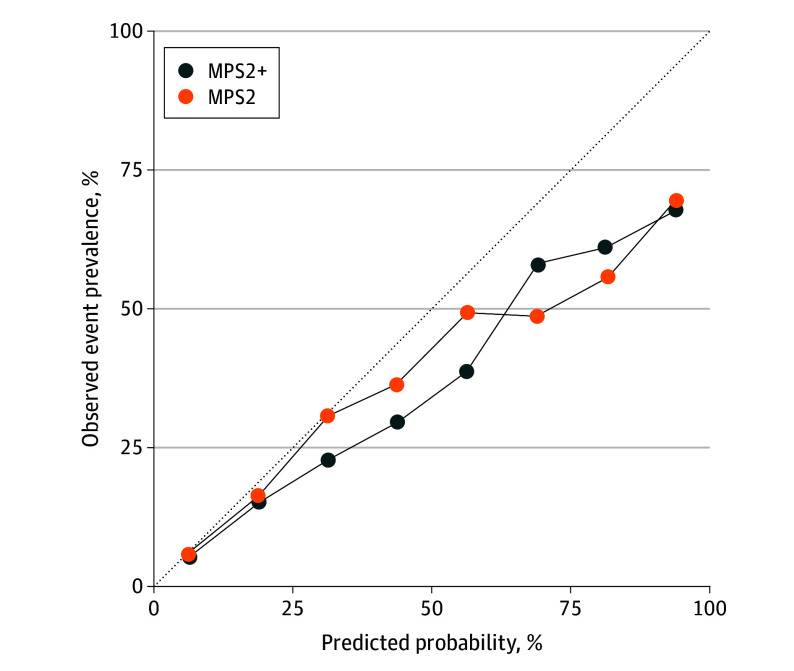
Calibration Curves for High-Grade Prostate Cancer for MyProstateScore 2.0 (MPS2) and MPS2 Plus Prostate Volume (MPS2+) in the External Validation Cohort

Of 813 patients in the validation cohort (eFigure 7 in Supplement 1), qPCR was successful in 743 (91.4%). The median (IQR) age was 62 (57-68) years, and the median (IQR) PSA level was 5.6 (4.1-8.0) ng/mL. A total of 95 men (12.8%) were Black and 648 (87.2%) were another race, and 247 men (33.2%) had a previous negative biopsy (Table 1). On study biopsy, 151 men (20.3%) had high-grade PCa. Median (IQR) MPS2 values were significantly higher in men with cancer of GG 2 or greater (0.44 [0.23-0.69]) than in men with negative biopsies (0.08 [0.03-0.19]; *P* < .001) and in men with cancer of GG 1 (0.20 [0.08-0.43]; *P* < .001) (Table 1; eFigure 8 in Supplement 1). Similarly, median (IQR) MPS2+ values were significantly higher in men with PCa of GG 2 or greater (0.54 [0.27-0.79]) relative to those with negative biopsies (0.08 [0.03-0.21]; *P* < .001) or those with cancer of GG 1 (0.25 [0.09-0.48]; *P* < .001). The AUC values for high-grade cancer were 0.60 (95% CI, 54.7-64.6) for PSA alone, 0.66 (95% CI, 61.1-70.7) for the Prostate Cancer Prevention Trial risk calculator, 0.77 (95% CI, 73.0-81.3) for PHI, 0.76 (95% CI, 71.9-80.3) for the derived multiplex 2-gene model, 0.72 (95% CI, 67.0-76.1) for the derived multiplex 3-gene model, and 0.74 (95% CI, 69.4-78.0) for MPS compared with 0.81 (95% CI, 76.9-84.6) for MPS2 and 0.82 (95% CI, 78.1-85.5) for MPS2+ (eFigure 9 in Supplement 1). The observed prevalence of high-grade cancer closely approximated MPS2 and MPS2+ predicted probabilities (Figure 2), reflecting good calibration. Critically, the models were particularly well-calibrated for predicted probabilities less than 30%, which are most clinically pertinent.

We assessed clinical consequences of prebiopsy biomarker testing. At a 95% sensitivity testing threshold, the proportions of unnecessary biopsies that would have been avoided using each test were 11% for PSA alone, 20% for the Prostate Cancer Prevention Trial risk calculator, 26% for PHI, 27% for the derived multiplex 2-gene model, 17% for the derived multiplex 3-gene model, and 23% for MPS compared with 37% for MPS2 and 41% for MPS2+. Full performance measures and the estimated numbers of unnecessary biopsies avoided per 1000 patients are listed in Table 2. Critically, MPS2 and MPS2+ each provided 99% sensitivity and 99% NPV for cancer of GG 3 or greater.

**Table 2.** Performance of Prostate-Specific Antigen (PSA) Alone, Prostate Cancer Prevention Trial Risk Calculator, Prostate Health Index (PHI), Derived Multiplex 2-Gene and 3-Gene Models, MyProstateScore (MPS), MPS2, and MPS2 Plus Prostate Volume (MPS2+) in the Validation Cohort

Model	%				Estimated unnecessary biopsies avoided per 1000 patients
	Sensitivity	Specificity	NPV	PPV	
**Overall (n = 743)**					
PSA	95	11	90	21	108
Prostate Cancer Prevention Trial risk calculator	95	20	94	23	198
PHI	95	26	96	25	258
Derived multiplex 2-gene model	95	27	96	25	270
Derived multiplex 3-gene model	95	17	94	23	171
MPS	95	23	94	24	230
MPS2	95	37	97	28	370
MPS2+	95	41	97	29	405
**Initial biopsy (n = 496)**					
PSA	95	15	89	29	152
Prostate Cancer Prevention Trial risk calculator	95	27	94	32	267
PHI	95	30	95	33	295
Derived multiplex 2-gene model	95	30	95	33	303
Derived multiplex 3-gene model	95	17	91	30	168
MPS	95	27	93	32	270
MPS2	95	35	95	35	347
MPS2+	95	42	96	37	419
**Repeat biopsy (n = 247)**					
PSA	94.4	15	97	8.0	148
Prostate Cancer Prevention Trial risk calculator	94.4	21	98	8.6	210
PHI	94.4	8.7	95	7.5	87
Derived multiplex 2-gene model	94.4	14	97	8.0	144
Derived multiplex 3-gene model	94.4	16	97	8.1	162
MPS	94.4	15	97	8.0	148
MPS2	94.4	46	99	12	462
MPS2+	94.4	51	99	13	511

Abbreviations: NPV, negative predictive value; PPV, positive predictive value.

#### Initial Biopsy Subpopulation

The initial biopsy population included 496 patients with a median (IQR) PSA level of 5.0 (3.8-6.6) ng/mL (eTable 5 in Supplement 1). On study biopsy, 133 (26.8%) had high-grade cancer. Using a 95% sensitivity threshold, the proportions of unnecessary biopsies avoided were 15% for PSA alone, 27% for the Prostate Cancer Prevention Trial risk calculator, 30% for PHI, 30% for the derived multiplex 2-gene model, 17% for the derived multiplex 3-gene model, and 27% for MPS compared with 35% for MPS2 (Table 2; eTable 6 in Supplement 1). Although patients undergoing initial biopsy often may not have prostate volume available, use of MPS2+ would have avoided 42% of unnecessary biopsies. Performance of MPS2 models with and without clinical factors are provided by subpopulation in eTables 7 and 8 in Supplement 1. An alternative initial biopsy model was developed in the initial biopsy population of the development cohort and similarly validated (eTables 9 and 10 and eFigure 10 in Supplement 1).

#### Repeat Biopsy Subpopulation

The repeat biopsy population included 247 men with median (IQR) PSA level of 7.2 (5.5-9.8) ng/mL, of which 18 (7.3%) were found to have high-grade cancer (eTable 5 in Supplement 1). At 95% sensitivity, the proportions of unnecessary biopsies that would have been avoided were 15% for PSA alone, 8.7% for PHI, 14% for the derived multiplex 2-gene model, 16% for the derived multiplex 3-gene model, 15% for MPS, 46% for MPS2, and 51% for MPS2+ (Table 2). Accordingly, MPS2 testing would have avoided approximately one-half of unnecessary biopsies while maintaining detection of 94.4% of high-grade cancers.

### DCA

**Figure 3.**
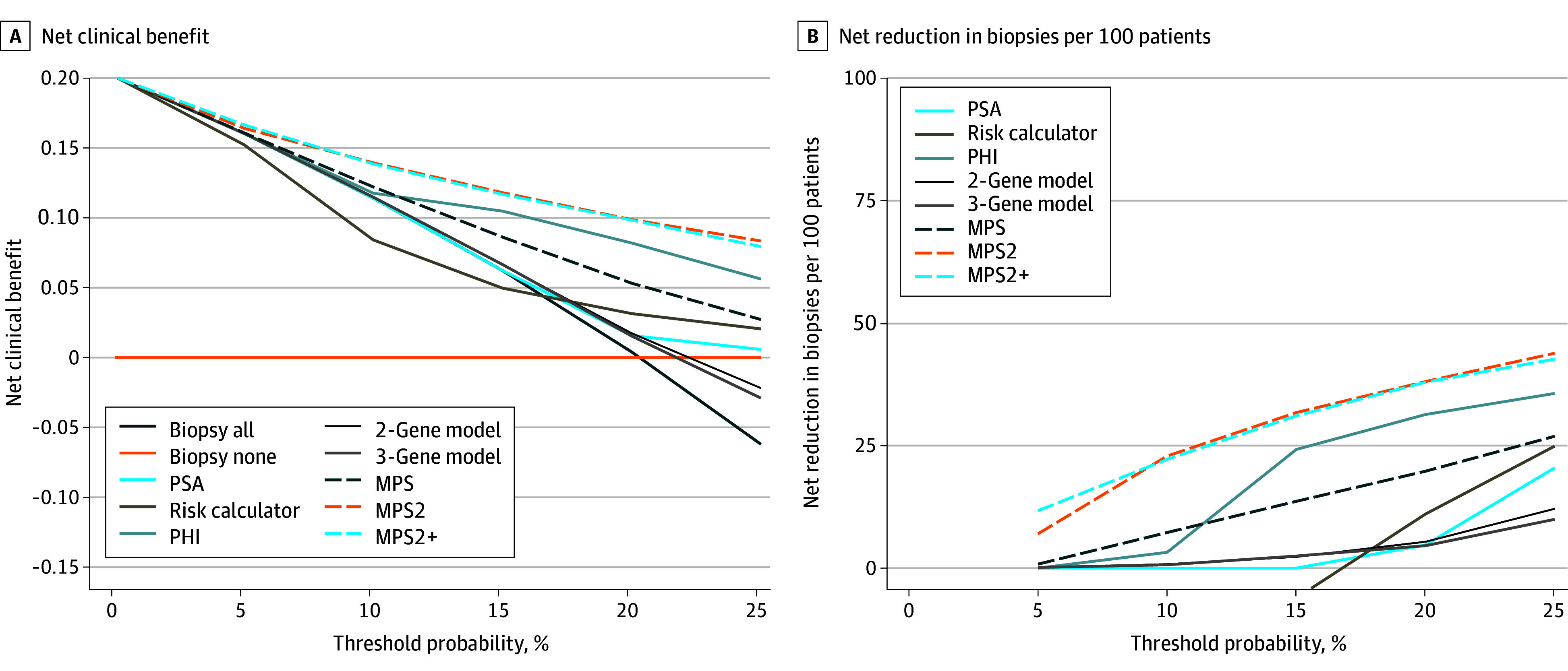
Decision Curve Analysis for High-Grade Prostate Cancer in the External Validation Cohort A, Decision curve analysis plots for net clinical benefit of prebiopsy testing with prostate-specific antigen (PSA) alone, the Prostate Cancer Prevention Trial risk calculator, Prostate Health Index (PHI), derived multiplex 2-gene model, derived multiplex 3-gene model, MyProstateScore (MPS), MPS2, and MPS2+ compared with baseline approaches of biopsy all or biopsy none. The threshold probability (x-axis) reflects how the patient and clinician value potential clinical outcomes. For example, a threshold probability of 5% applies to patients that would choose to pursue biopsy if their risk of high-grade cancer is 5% or higher. For high-grade prostate cancer, a 5% threshold probability represents a risk-averse population, such as younger men with a long life expectancy. At a practice level, this implies that the clinician would be willing to perform as many as 20 biopsies to detect an additional high-grade cancer. At the other end of the spectrum, a threshold probability of 20% applies to patients that would choose to pursue biopsy only if their risk of high-grade cancer was 20% or greater. Such a population strongly values avoiding biopsy and is willing to accept a higher risk of delayed detection of high-grade cancer. The unit of net benefit (y-axis) is true positives. A net benefit of 0.15 is equivalent to an approach in which 15 patients per 100 are directed to biopsy based on use of the test, and all 15 patients are found to have high-grade cancer. As illustrated in the figure, the MPS2 and MPS2+ models provided the highest net benefit across the range of clinically pertinent threshold probabilities (5% to 20%). B, Decision curve analysis plots illustrating the net reduction in biopsies performed per 100 patients without missing a single diagnosis of cancer of grade group 2 or greater based on prebiopsy testing with PSA alone, the Prostate Cancer Prevention Trial risk calculator, PHI, the derived multiplex 2-gene model, the derived multiplex 3-gene model, MPS, MPS2, and MPS2+ compared with a baseline approach of biopsying all patients. The MPS2 and MPS2+ models provided the largest net reduction in biopsies performed across clinically pertinent threshold probabilities.

DCA was used to evaluate the net benefit of biomarker testing relative to performing biopsy in all patients and performing no biopsies. Across the clinically pertinent threshold probabilities spanning 5% to 20%, use of the MPS2 models would have provided the highest net clinical benefit across all tests (Figure 3A). Expressing benefit as net reduction in unnecessary biopsies, use of the MPS2 models would have provided the greatest net reduction in unnecessary biopsies without failing to biopsy a single patient with high-grade cancer (Figure 3B).

## Discussion

Acknowledging the indolent nature of low-grade PCa, contemporary guidelines emphasize a narrowed diagnostic focus on high-grade cancers.^5,6,44^ Existing biomarkers expressed by all PCa—including low-grade, indolent tumors—therefore offer limited potential to selectively detect high-grade disease. Translating sequencing-based discovery to an expandable qPCR platform, we developed and validated a new urinary test incorporating 17 markers of cancer, and—for the first time, to our knowledge—novel markers uniquely overexpressed by high-grade cancers relative to low-grade cancers. On validation, MPS2 testing with 95% sensitivity for high-grade cancer had 95% to 99% NPV and 35% to 51% specificity across subgroups. For individual patients, NPVs approaching 100% provide clear guidance for confident decision-making. For clinicians, uniform use of MPS2 could avoid unnecessary biopsies while preserving immediate detection of 95% of cancers of GG 2 or greater diagnosed using the biopsy all approach. Critically, MPS2 had 99% sensitivity and 99% NPV for cancers of GG 3 or greater, meaning the rare false-negative MPS2 results were almost uniformly more favorable cancers of GG 2 least likely to metastasize.

The 2023 NCCN guidelines for PCa early detection propose consideration of prebiopsy risk stratification with validated biomarker tests, including PHI, SelectMDx, 4Kscore, EPI, MPS, and IsoPSA.^5^ These tests have consistently outperformed PSA alone, with aggregate data approximating 90% to 95% sensitivity and 30% to 40% specificity for high-grade cancer.^13,28,32^ However, heterogeneity of published data and a lack of head-to-head comparisons have precluded recommendations of any particular testing approach.^45^ Using an NCI cohort clinically indicated for biomarker testing, we directly compared the new 18-gene test for high-grade PCa with existing guideline-endorsed tests. Broadly, AUC values for MPS2 models were associated with meaningful improvement compared with currently available options. Using a testing approach with 95% sensitivity for high-grade cancer, MPS2 would have meaningfully reduced unnecessary biopsies performed relative to other tests. These data support use of MPS2 to mitigate the potential harms of screening while preserving its long-term benefits.

Patients with a prior negative biopsy pose a unique challenge.^40^ Because most patients undergo initial biopsy due to PSA elevation, the value of repeated PSA testing is particularly limited in this population.^46^ In one prior study, among 229 patients undergoing repeat biopsies, the EPI test provided 82% sensitivity and 27% specificity for high-grade cancer.^47^ Among 268 patients undergoing repeat biopsies, MPS provided 100% sensitivity and 23% specificity.^48^ In the current analysis including 247 patients, at 94.4% sensitivity, MPS2+ provided 51% specificity, compared with 8.7% with PHI, 14% with the derived multiplex 2-gene model, 16% with the derived multiplex 3-gene model, and 15% with MPS. While striking, these findings are plausible, as most current assays include PSA and PSA isoforms, underscoring a continued dependence on PSA. Second, existing assays measure 3 or fewer non-PSA markers. Given the multiple pathways driving lethal PCa,^14,15^ it is difficult to conceive that most aggressive cancers would overexpress one of so few markers early in the disease course. By capturing 17 cancer-associated, PSA-independent markers, MPS2 provides roughly 5-fold the breadth of previous tests and offers promise of a new generation of biomarkers not reliant on PSA.

The ideal diagnostic test has been described as safe, accurate, available, and actionable and providing a favorable benefit-to-harm ratio.^49,50^ While PSA alone offers favorable practical attributes, its lack of cancer specificity has driven the need for a complementary test to improve screening outcomes.^4^ While prebiopsy mpMRI improves detection of high-grade cancer in men with positive findings on mpMRI,^7,51^ data describing the use of negative mpMRI findings to rule out significant cancer merit concern. Findings from a statewide collaborative revealed an NPV of only 77% across diverse settings.^8^ Even at experienced centers, subjective MRI interpretation yields significant variability, with NPVs as low as 63% at one center and 40% among individual radiologists.^9,10^ Moreover, MRI bears an extensive time and resource burden, is not widely available in community settings, and is not an option for some patients, posing critical barriers to widespread use.^11,12^ While a valuable component of the diagnostic armamentarium, practical limitations and suboptimal rule-out performance suggest MRI may be best used later in the diagnostic pathway, eg, to improve the yield of biopsy in men most likely to benefit from invasive testing.

The accuracy of MPS2 offers potential for straightforward application at the primary care level (ie, negative test rules out high-grade disease; positive test prompts specialist referral). For specialists, providing patients with early noninvasive molecular tumor data^52,53,54,55^ could enable more informed, individualized cancer care. For example, in patients indicated for biopsy, the relationship of tumor subtypes with MRI visibility could help identify patients likely to benefit from prebiopsy mpMRI and those better served by immediate biopsy.^56^ In men with PCa of GG 1, high-grade markers could signal the presence of occult aggressive tumors, while their absence could obviate the need for scheduled surveillance biopsies.^57^ Finally, while biopsy and tissue-based assays rely on the specific tumor foci sampled,^58,59^ urine provides a comprehensive assessment of prostatic gene expression—an ideal complement to mitigate the sampling limitations of biopsy.

### Limitations

The current study has limitations. For one, there was limited racial diversity in the study population. Thus, it is unclear how our findings could differ in Black men, and we are currently pursuing analyses to ensure optimal testing for all patients. Second, the reference standard was systematic biopsy, which is subject to undersampling that could increase NPV and decrease positive predictive value relative to surgical pathology.^60,61,62^ Nonetheless, sampling misclassification would be expected to impact all tests equally, and we uniquely performed head-to-head comparison of MPS2 with existing biomarker tests. Furthermore, we repeated model development in patients with more definitive pathologic data (eg, radical prostatectomy), and prostatectomy-derived MPS2 models did not differ substantially (eTable 11 in Supplement 1). Notably, the current analysis used the Prostate Cancer Prevention Trial risk calculator due to its extensive validation and recognition by clinicians^63^; other risk calculators could have performed differently.^64^

We acknowledge the limitations of deriving molecular models developed on other platforms. Although the derived multiplex models capture the components of other commercially available tests, these models should not be interpreted as equivalent to the commercial assays, just as no conclusions can be drawn regarding biomarkers not assessed. Still, external comparison of a newly validated test with guideline-endorsed tests has not previously been performed, to our knowledge, and the 18-gene test would have yielded clinically meaningful improvement in accuracy for high-grade PCa relative to current testing options. While encouraging, these findings do not rule out disparate findings in additional cohorts. Moreover, the 95% sensitivity threshold is a single data point that, while illustrative and clinically applicable, may not be ideal for all populations; decision curves presented herein provide a greater breadth of information regarding utility. Finally, this study population was not suitable for comparing biomarkers with mpMRI, which remains a critical knowledge gap. We are currently conducting a prospective multicenter trial for this assessment.^65^ Regardless, the externally validated performance of MPS2 supports its effectiveness in accurately ruling out the need for mpMRI and biopsy altogether. Additional studies are needed to corroborate these data and confirm the observed positive impact of MPS2 testing on longer-term outcomes.

## Conclusions

In this study, within an external validation population referred for prostate biopsy, an 18-gene urinary test had higher diagnostic accuracy for high-grade PCa beyond currently available testing options. Clinically, use of this test would have safely avoided unnecessary additional testing with imaging or biopsy in 35% to 51% of patients while maintaining high sensitivity for high-grade cancers that stand to benefit from early detection. These findings suggest that use of the test in patients with elevated PSA levels can reduce the potential harms of prostate cancer screening while preserving its long-term benefits.

## Notes

Supplement 1. **eTable 1.** RNAseq-Based Marker Nomination Criteria **eTable 2.** Indications for Prostate Biopsy in the External Validation Cohort **eTable 3.** Frequency of Inclusion and Cumulative Importance of the 17 Most Informative Markers Across 40 Elastic Net Models Assessed in Development **eTable 4.** Model Coefficients for the MPS2 and MPS2+ Models **eTable 5.** Characteristics of the NCI EDRN External Validation Population Stratified by Previous Biopsy Status **eTable 6.** Performance Measure Calculations for High-Grade Cancer using PSA Alone, Prostate Cancer Prevention Trial Risk Calculator, Prostate Health Index (PHI), Derived Multiplex 2-Gene and 3-Gene Models, MyProstateScore (MPS), MPS2, and MPS2 Plus Prostate Volume (MPS2+) in the EDRN Validation Cohort **eTable 7.** Clinical Performance of High Sensitivity MPS2 Threshold Values in the Initial Biopsy Subpopulation of the External Validation Population **eTable 8.** Clinical Performance of High Sensitivity MPS2 Threshold Values in the Repeat Biopsy Subpopulation of the External Validation Population **eTable 9.** Characteristics of the Secondary Development Cohort for Patients Undergoing Initial Biopsy at U-M **eTable 10.** Clinical Performance of Secondary Initial Biopsy (iMPS2) Models in the Initial Biopsy Subpopulation of the External Validation Cohort (n = 496) **eTable 11.** Model Coefficients from MPS2 Redevelopment Using Maximum Grade Group From All Biopsy and Surgical Specimens **eFigure 1.** Heatmap Illustrating Differential Expression of RNAseq Target Genes **eFigure 2.** Box and Dot Plots of Gene Expression in Benign, Low-Grade Cancer, and High-Grade Cancer Tissue in the Discovery Set **eFigure 3.** Box and Dot Plots of Gene Expression in Benign vs Prostate Cancer Tissue in the Discovery Set **eFigure 4.** Flow Diagram of the University of Michigan Development Cohort **eFigure 5.** Postcalibration Curves of MPS2 and MPS2+ in the Development Cohort **eFigure 6.** MPS2 and MPS2+ Cross-Validated Area Under the ROC Curves for High-Grade Prostate Cancer in the Development Cohort **eFigure 7.** Flow Diagram of the NCI EDRN External Validation Cohort **eFigure 8.** MPS2 Values by Biopsy Pathology in the External Validation Cohort **eFigure 9.** MPS2 and MPS2+ Area Under the ROC Curves for High-Grade Prostate Cancer in the External Validation Cohort **eFigure 10.** Postcalibration Curves of iMPS2 and iMPS2+ in the External Validation Cohort
eReferences.

Supplement 2. **eTable.** Characterization of 58 724 Gene Targets Assessed by RNAseq

Supplement 3. **eTable.** Characterization of the 54-Marker Candidate Panel

Supplement 4. **Group Information.** EDRN-PCA3 Study Group

Supplement 5. Data Sharing Statement

## References

[1] Kocarnik JM, Compton K, Dean FE, ; Global Burden of Disease 2019 Cancer Collaboration. Cancer incidence, mortality, years of life lost, years lived with disability, and disability-adjusted life years for 29 cancer groups from 2010 to 2019: a systematic analysis for the Global Burden of Disease Study 2019. JAMA Oncol. 2022;8(3):420-444. doi:10.1001/jamaoncol.2021.6987 34967848 PMC8719276

[2] Hugosson J, Roobol MJ, Månsson M, ; ERSPC investigators. A 16-yr follow-up of the European Randomized Study of Screening for Prostate Cancer. Eur Urol. 2019;76(1):43-51. doi:10.1016/j.eururo.2019.02.009 30824296 PMC7513694

[3] Frånlund M, Månsson M, Godtman RA, . Results from 22 years of followup in the Göteborg randomized population-based prostate cancer screening trial. J Urol. 2022;208(2):292-300. doi:10.1097/JU.0000000000002696 35422134 PMC9275849

[4] Fenton JJ, Weyrich MS, Durbin S, Liu Y, Bang H, Melnikow J. Prostate-specific antigen-based screening for prostate cancer: evidence report and systematic review for the US Preventive Services Task Force. JAMA. 2018;319(18):1914-1931. doi:10.1001/jama.2018.3712 29801018

[5] National Comprehensive Cancer Network. Prostate cancer early detection (version 1.2023). Accessed May 19, 2023. https://www.nccn.org/professionals/physician_gls/pdf/prostate_detection.pdf 10.6004/jnccn.2023.0014 36898362

[6] Wei JT, Barocas D, Carlsson S, . Early detection of prostate cancer: AUA/SUO guideline part i: prostate cancer screening. J Urol. 2023;210(1):46-53. doi:10.1097/JU.0000000000003491 37096582 PMC11060750

[7] Ahdoot M, Wilbur AR, Reese SE, . MRI-targeted, systematic, and combined biopsy for prostate cancer diagnosis. N Engl J Med. 2020;382(10):917-928. doi:10.1056/NEJMoa1910038 32130814 PMC7323919

[8] Zhu AAS, DiBianco JM, Qi J, . PD38-01 Negative predictive value of prostate MRI in real world practice: results from a statewide surgical collaboration. J Urol. 2023;209(4):e993. doi:10.1097/JU.0000000000003336.01

[9] Sathianathen NJ, Omer A, Harriss E, . Negative predictive value of multiparametric magnetic resonance imaging in the detection of clinically significant prostate cancer in the prostate imaging reporting and data system era: a systematic review and meta-analysis. Eur Urol. 2020;78(3):402-414. doi:10.1016/j.eururo.2020.03.048 32444265

[10] Sonn GA, Fan RE, Ghanouni P, . Prostate magnetic resonance imaging interpretation varies substantially across radiologists. Eur Urol Focus. 2019;5(4):592-599. doi:10.1016/j.euf.2017.11.010 29226826

[11] Jiao B, Gulati R, Hendrix N, . Economic evaluation of urine-based or magnetic resonance imaging reflex tests in men with intermediate prostate-specific antigen levels in the United States. Value Health. 2021;24(8):1111-1117. doi:10.1016/j.jval.2021.02.009 34372976 PMC8358184

[12] Borregales LD, DeMeo G, Gu X, . Grade migration of prostate cancer in the United States during the last decade. J Natl Cancer Inst. 2022;114(7):1012-1019. doi:10.1093/jnci/djac066 35348709 PMC9275764

[13] Eyrich NW, Morgan TM, Tosoian JJ. Biomarkers for detection of clinically significant prostate cancer: contemporary clinical data and future directions. Transl Androl Urol. 2021;10(7):3091-3103. doi:10.21037/tau-20-1151 34430413 PMC8350244

[14] Abeshouse A, Ahn J, Akbani R, ; Cancer Genome Atlas Research Network. The molecular taxonomy of primary prostate cancer. Cell. 2015;163(4):1011-1025. doi:10.1016/j.cell.2015.10.025 26544944 PMC4695400

[15] Fraser M, Sabelnykova VY, Yamaguchi TN, . Genomic hallmarks of localized, non-indolent prostate cancer. Nature. 2017;541(7637):359-364. doi:10.1038/nature20788 28068672

[16] Prensner JR, Iyer MK, Balbin OA, . Transcriptome sequencing across a prostate cancer cohort identifies PCAT-1, an unannotated lincRNA implicated in disease progression. Nat Biotechnol. 2011;29(8):742-749. doi:10.1038/nbt.1914 21804560 PMC3152676

[17] Iyer MK, Niknafs YS, Malik R, . The landscape of long noncoding RNAs in the human transcriptome. Nat Genet. 2015;47(3):199-208. doi:10.1038/ng.3192 25599403 PMC4417758

[18] Bossuyt PM, Reitsma JB, Bruns DE, ; STARD Group. STARD 2015: an updated list of essential items for reporting diagnostic accuracy studies. BMJ. 2015;351:h5527. doi:10.1136/bmj.h5527 26511519 PMC4623764

[19] Tomlins SA, Day JR, Lonigro RJ, . Urine TMPRSS2:ERG plus PCA3 for individualized prostate cancer risk assessment. Eur Urol. 2016;70(1):45-53. doi:10.1016/j.eururo.2015.04.039 25985884 PMC4644724

[20] Wei JT, Feng Z, Partin AW, . Can urinary PCA3 supplement PSA in the early detection of prostate cancer? J Clin Oncol. 2014;32(36):4066-4072. doi:10.1200/JCO.2013.52.8505 25385735 PMC4265117

[21] Thompson IM, Ankerst DP, Chi C, . Assessing prostate cancer risk: results from the Prostate Cancer Prevention Trial. J Natl Cancer Inst. 2006;98(8):529-534. doi:10.1093/jnci/djj131 16622122

[22] Ankerst DP, Till C, Boeck A, . The impact of prostate volume, number of biopsy cores and American Urological Association symptom score on the sensitivity of cancer detection using the Prostate Cancer Prevention Trial risk calculator. J Urol. 2013;190(1):70-76. doi:10.1016/j.juro.2012.12.108 23313212 PMC3708069

[23] Roobol MJ, Schröder FH, Hugosson J, . Importance of prostate volume in the European Randomised Study of Screening for Prostate Cancer (ERSPC) risk calculators: results from the Prostate Biopsy Collaborative Group. World J Urol. 2012;30(2):149-155. doi:10.1007/s00345-011-0804-y 22203238 PMC3321270

[24] Vergouwe Y, Nieboer D, Oostenbrink R, . A closed testing procedure to select an appropriate method for updating prediction models. Stat Med. 2017;36(28):4529-4539. doi:10.1002/sim.7179 27891652

[25] Moons KG, Altman DG, Reitsma JB, . Transparent Reporting of a Multivariable Prediction Model for Individual Prognosis or Diagnosis (TRIPOD): explanation and elaboration. Ann Intern Med. 2015;162(1):W1-73. doi:10.7326/M14-0698 25560730

[26] Lillard JW Jr, Moses KA, Mahal BA, George DJ. Racial disparities in Black men with prostate cancer: a literature review. Cancer. 2022;128(21):3787-3795. doi:10.1002/cncr.34433 36066378 PMC9826514

[27] Johnson JA, Moore B, Hwang EK, Hickner A, Yeo H. The accuracy of race & ethnicity data in US based healthcare databases: a systematic review. Am J Surg. 2023;226(4):463-470. doi:10.1016/j.amjsurg.2023.05.011 37230870

[28] Sanda MG, Feng Z, Howard DH, ; and the EDRN-PCA3 Study Group. Association between combined TMPRSS2:ERG and PCA3 RNA urinary testing and detection of aggressive prostate cancer. JAMA Oncol. 2017;3(8):1085-1093. doi:10.1001/jamaoncol.2017.0177 28520829 PMC5710334

[29] Robin X, Turck N, Hainard A, . pROC: an open-source package for R and S+ to analyze and compare ROC curves. BMC Bioinformatics. 2011;12:77. doi:10.1186/1471-2105-12-77 21414208 PMC3068975

[30] Tosoian JJ, Trock BJ, Morgan TM, . Use of the MyProstateScore Test to rule out clinically significant cancer: validation of a straightforward clinical testing approach. J Urol. 2021;205(3):732-739. doi:10.1097/JU.0000000000001430 33080150 PMC8189629

[31] de la Calle C, Patil D, Wei JT, . Multicenter evaluation of the prostate health index to detect aggressive prostate cancer in biopsy naïve men. J Urol. 2015;194(1):65-72. doi:10.1016/j.juro.2015.01.091 25636659 PMC4696043

[32] McKiernan J, Donovan MJ, O’Neill V, . A novel urine exosome gene expression assay to predict high-grade prostate cancer at initial biopsy. JAMA Oncol. 2016;2(7):882-889. doi:10.1001/jamaoncol.2016.0097 27032035

[33] Parekh DJ, Punnen S, Sjoberg DD, . A multi-institutional prospective trial in the USA confirms that the 4Kscore accurately identifies men with high-grade prostate cancer. Eur Urol. 2015;68(3):464-470. doi:10.1016/j.eururo.2014.10.021 25454615

[34] Loeb S, Sanda MG, Broyles DL, . The Prostate Health Index selectively identifies clinically significant prostate cancer. J Urol. 2015;193(4):1163-1169. doi:10.1016/j.juro.2014.10.121 25463993 PMC4404198

[35] Haese A, Trooskens G, Steyaert S, . Multicenter optimization and validation of a 2-gene mRNA urine test for detection of clinically significant prostate cancer before initial prostate biopsy. J Urol. 2019;202(2):256-263. doi:10.1097/JU.0000000000000293 31026217

[36] Assel M, Sjoberg D, Elders A, . Guidelines for reporting of statistics for clinical research in urology. Eur Urol. 2019;75(3):358-367. doi:10.1016/j.eururo.2018.12.014 30580902 PMC6391870

[37] US Centers for Medicare & Medicaid Services. Biomarker testing for prostate cancer diagnosis. Accessed March 23, 2020. https://www.cms.gov/medicare-coverage-database/view/lcd.aspx?LCDId=37733

[38] Nordström T, Vickers A, Assel M, Lilja H, Grönberg H, Eklund M. Comparison between the four-kallikrein panel and Prostate Health Index for predicting prostate cancer. Eur Urol. 2015;68(1):139-146. doi:10.1016/j.eururo.2014.08.010 25151013 PMC4503229

[39] ElShafei A, Nyame Y, Kara O, . More favorable pathological outcomes in men with low risk prostate cancer diagnosed on repeat versus initial transrectal ultrasound guided prostate biopsy. J Urol. 2016;195(6):1767-1772. doi:10.1016/j.juro.2015.12.079 26724397

[40] Kearns J, Lin D. Utilizing biomarkers in patients with prior negative prostate biopsy. In: Change SS, Cookson MS, eds. Prostate Cancer: Clinical Case Scenarios. Springer International Publishing; 2018:43-52.

[41] Tan N, Lane BR, Li J, Moussa AS, Soriano M, Jones JS. Prostate cancers diagnosed at repeat biopsy are smaller and less likely to be high grade. J Urol. 2008;180(4):1325-1329. doi:10.1016/j.juro.2008.06.022 18707706

[42] Vickers AJ, van Calster B, Steyerberg EW. A simple, step-by-step guide to interpreting decision curve analysis. Diagn Progn Res. 2019;3:18. doi:10.1186/s41512-019-0064-7 31592444 PMC6777022

[43] Vickers AJ, Van Calster B, Steyerberg EW. Net benefit approaches to the evaluation of prediction models, molecular markers, and diagnostic tests. BMJ. 2016;352:i6. doi:10.1136/bmj.i6 26810254 PMC4724785

[44] European Association of Urologists Guidelines Office. Prostate cancer. Accessed December 1, 2023. https://uroweb.org/guidelines/prostate-cancer

[45] Narayan VM. A critical appraisal of biomarkers in prostate cancer. World J Urol. 2020;38(3):547-554. doi:10.1007/s00345-019-02759-x 30993424

[46] Nordström T, Adolfsson J, Grönberg H, Eklund M. Repeat prostate-specific antigen tests before prostate biopsy decisions. J Natl Cancer Inst. 2016;108(12):djw165. doi:10.1093/jnci/djw165 27418620

[47] McKiernan J, Noerholm M, Tadigotla V, . A urine-based exosomal gene expression test stratifies risk of high-grade prostate cancer in men with prior negative prostate biopsy undergoing repeat biopsy. BMC Urol. 2020;20(1):138. doi:10.1186/s12894-020-00712-4 32873277 PMC7466797

[48] Tosoian JJ, Sessine MS, Trock BJ, . MyProstateScore in men considering repeat biopsy: validation of a simple testing approach. Prostate Cancer Prostatic Dis. 2023;26(3):563-567. doi:10.21203/rs.3.rs-1728404/v1 36585434 PMC10310885

[49] UK National Screening Committee. Criteria for a population screening programme. Accessed December 1, 2023. https://www.gov.uk/government/publications/evidence-review-criteria-national-screening-programmes/criteria-for-appraising-the-viability-effectiveness-and-appropriateness-of-a-screening-programme

[50] Wilson JMG, Jungner G. Principles and practice of screening for disease. Accessed December 1, 2023. https://apps.who.int/iris/bitstream/handle/10665/37650/WHO_PHP_34.pdf?sequence=17&isAllowed=y

[51] Bloom JB, Daneshvar MA, Lebastchi AH, . Risk of adverse pathology at prostatectomy in the era of MRI and targeted biopsies; rethinking active surveillance for intermediate risk prostate cancer patients. Urol Oncol. 2021;39(10):729.e1-729.e6. doi:10.1016/j.urolonc.2021.02.018 33736975

[52] Nakanishi H, Groskopf J, Fritsche HA, . PCA3 molecular urine assay correlates with prostate cancer tumor volume: implication in selecting candidates for active surveillance. J Urol. 2008;179(5):1804-1809. doi:10.1016/j.juro.2008.01.013 18353398

[53] Prensner JR, Zhao S, Erho N, . RNA biomarkers associated with metastatic progression in prostate cancer: a multi-institutional high-throughput analysis of SChLAP1. Lancet Oncol. 2014;15(13):1469-1480. doi:10.1016/S1470-2045(14)71113-1 25456366 PMC4559342

[54] Young A, Palanisamy N, Siddiqui J, . Correlation of urine TMPRSS2:ERG and PCA3 to ERG+ and total prostate cancer burden. Am J Clin Pathol. 2012;138(5):685-696. doi:10.1309/AJCPU7PPWUPYG8OH 23086769 PMC3597433

[55] Hendriks R, Dijkstra S, Cornel EB, . Elevated HOXC6/DLX1 mRNA biomarker levels in urine to help select patients at increased risk for high-grade prostate cancer detection upon prostate biopsy. J Clin Oncol. 2016;34(2)(suppl):31. doi:10.1200/jco.2016.34.2_suppl.31

[56] Salami SS, Kaplan JB, Nallandhighal S, . Biologic significance of magnetic resonance imaging invisibility in localized prostate cancer. JCO Precis Oncol. 2019;3:PO.19.00054. Published online June 12, 2019. doi:10.1200/PO.19.00054 32914029 PMC7446477

[57] Klein EA, Cooperberg MR, Magi-Galluzzi C, . A 17-gene assay to predict prostate cancer aggressiveness in the context of Gleason grade heterogeneity, tumor multifocality, and biopsy undersampling. Eur Urol. 2014;66(3):550-560. doi:10.1016/j.eururo.2014.05.004 24836057

[58] Salami SS, Hovelson DH, Kaplan JB, . Transcriptomic heterogeneity in multifocal prostate cancer. JCI Insight. 2018;3(21):e123468. doi:10.1172/jci.insight.123468 30385730 PMC6238741

[59] Wei L, Wang J, Lampert E, . Intratumoral and intertumoral genomic heterogeneity of multifocal localized prostate cancer impacts molecular classifications and genomic prognosticators. Eur Urol. 2017;71(2):183-192. doi:10.1016/j.eururo.2016.07.008 27451135 PMC5906059

[60] Moussa AS, Kattan MW, Berglund R, Yu C, Fareed K, Jones JS. A nomogram for predicting upgrading in patients with low- and intermediate-grade prostate cancer in the era of extended prostate sampling. BJU Int. 2010;105(3):352-358. doi:10.1111/j.1464-410X.2009.08778.x 19681898

[61] Epstein JI, Feng Z, Trock BJ, Pierorazio PM. Upgrading and downgrading of prostate cancer from biopsy to radical prostatectomy: incidence and predictive factors using the modified Gleason grading system and factoring in tertiary grades. Eur Urol. 2012;61(5):1019-1024. doi:10.1016/j.eururo.2012.01.050 22336380 PMC4659370

[62] Bullock N, Simpkin A, Fowler S, Varma M, Kynaston H, Narahari K. Pathological upgrading in prostate cancer treated with surgery in the United Kingdom: trends and risk factors from the British Association of Urological Surgeons Radical Prostatectomy Registry. BMC Urol. 2019;19(1):94. doi:10.1186/s12894-019-0526-9 31623595 PMC6798468

[63] Bandala-Jacques A, Castellanos Esquivel KD, Pérez-Hurtado F, Hernández-Silva C, Reynoso-Noverón N. Prostate cancer risk calculators for healthy populations: systematic review. JMIR Cancer. 2021;7(3):e30430. doi:10.2196/30430 34477564 PMC8449298

[64] Ankerst DP, Straubinger J, Selig K, . A contemporary prostate biopsy risk calculator based on multiple heterogeneous cohorts. Eur Urol. 2018;74(2):197-203. doi:10.1016/j.eururo.2018.05.003 29778349 PMC6082177

[65] EDRN Prostate MRI Biomarker Study (P-MRI). ClinicalTrials.gov identifier: NCT03784924. Updated February 23, 2024. Accessed December 1, 2023. https://ClinicalTrials.gov/show/NCT03784924

