# Supplementary material for: Development and Validation of an 18-Gene Urine Test for High-Grade Prostate Cancer
Source: JAMA Oncol. 2024 Apr 18;10(6):726–36. doi: 10.1001/jamaoncol.2024.0455 (PMC11190811; doi:10.1001/jamaoncol.2024.0455)
Supplement: Supplement 1. — eTable 1. RNAseq-Based Marker Nomination Criteria eTable 2. Indications for Prostate Biopsy in the External Validation Cohort eTable 3. Frequency of Inclusion and Cumulative Importance of the 17 Most Informative Markers Across 40 Elastic Net Models Assessed in Development eTable 4. Model Coefficients for the MPS2 and MPS2+ Models eTable 5. Characteristics of the NCI EDRN External Validation Population Stratified by Previous Biopsy Status eTable 6. Performance Measure Calculations for High-Grade Cancer using PSA Alone, Prostate Cancer Prevention Trial Risk Calculator, Prostate Health Index (PHI), Derived Multiplex 2-Gene and 3-Gene Models, MyProstateScore (MPS), MPS2, and MPS2 Plus Prostate Volume (MPS2+) in the EDRN Validation Cohort eTable 7. Clinical Performance of High Sensitivity MPS2 Threshold Values in the Initial Biopsy Subpopulation of the External Validation Population eTable 8. Clinical Performance of High Sensitivity MPS2 Threshold Values in the Repeat Biopsy Subpopulation of the External Validation Population eTable 9. Characteristics of the Secondary Development Cohort for Patients Undergoing Initial Biopsy at U-M eTable 10. Clinical Performance of Secondary Initial Biopsy (iMPS2) Models in the Initial Biopsy Subpopulation of the External Validation Cohort (n = 496) eTable 11. Model Coefficients from MPS2 Redevelopment Using Maximum Grade Group From All Biopsy and Surgical Specimens eFigure 1. Heatmap Illustrating Differential Expression of RNAseq Target Genes eFigure 2. Box and Dot Plots of Gene Expression in Benign, Low-Grade Cancer, and High-Grade Cancer Tissue in the Discovery Set eFigure 3. Box and Dot Plots of Gene Expression in Benign vs Prostate Cancer Tissue in the Discovery Set eFigure 4. Flow Diagram of the University of Michigan Development Cohort eFigure 5. Postcalibration Curves of MPS2 and MPS2+ in the Development Cohort eFigure 6. MPS2 and MPS2+ Cross-Validated Area Under the ROC Curves for High-Grade Prostate Cancer in the Development C [file jamaoncol-e240455-s001.pdf]

Supplemental Online Content

Tosoian JJ, Zhang Y, Xiao L, et al; EDRN-PCA3 Study Group. Development and validation of an 18-gene urine test for high-grade prostate cancer. *JAMA Oncol*. Published online April 18, 2024. doi:10.1001/jamaoncol.2024.0455

eTable 1. RNAseq-Based Marker Nomination Criteria
eTable 2. Indications for Prostate Biopsy in the External Validation Cohort
eTable 3. Frequency of Inclusion and Cumulative Importance of the 17 Most Informative Markers Across 40 Elastic Net Models Assessed in Development
eTable 4. Model Coefficients for the MPS2 and MPS2+ Models
eTable 5. Characteristics of the NCI EDRN External Validation Population Stratified by Previous Biopsy Status
eTable 6. Performance Measure Calculations for High-Grade Cancer using PSA Alone, Prostate Cancer Prevention Trial Risk Calculator, Prostate Health Index (PHI), Derived Multiplex 2-Gene and 3-Gene Models, MyProstateScore (MPS), MPS2, and MPS2 Plus Prostate Volume (MPS2+) in the EDRN Validation Cohort
eTable 7. Clinical Performance of High Sensitivity MPS2 Threshold Values in the Initial Biopsy Subpopulation of the External Validation Population
eTable 8. Clinical Performance of High Sensitivity MPS2 Threshold Values in the Repeat Biopsy Subpopulation of the External Validation Population
eTable 9. Characteristics of the Secondary Development Cohort for Patients Undergoing Initial Biopsy at U-M
eTable 10. Clinical Performance of Secondary Initial Biopsy (iMPS2) Models in the Initial Biopsy Subpopulation of the External Validation Cohort (n = 496)
eTable 11. Model Coefficients from MPS2 Redevelopment Using Maximum Grade Group From All Biopsy and Surgical Specimens
eFigure 1. Heatmap Illustrating Differential Expression of RNAseq Target Genes
eFigure 2. Box and Dot Plots of Gene Expression in Benign, Low-Grade Cancer, and High-Grade Cancer Tissue in the Discovery Set
eFigure 3. Box and Dot Plots of Gene Expression in Benign vs Prostate Cancer Tissue in the Discovery Set
eFigure 4. Flow Diagram of the University of Michigan Development Cohort
eFigure 5. Postcalibration Curves of MPS2 and MPS2+ in the Development Cohort
eFigure 6. MPS2 and MPS2+ Cross-Validated Area Under the ROC Curves for High-Grade Prostate Cancer in the Development Cohort
eFigure 7. Flow Diagram of the NCI EDRN External Validation Cohort
eFigure 8. MPS2 Values by Biopsy Pathology in the External Validation Cohort
eFigure 9. MPS2 and MPS2+ Area Under the ROC Curves for High-Grade Prostate Cancer in the External Validation Cohort
eFigure 10. Postcalibration Curves of iMPS2 and iMPS2+ in the External Validation Cohort
eReferences.

This supplementary material has been provided by the authors to give readers additional information about their work.

© 2024 Tosoian JJ et al. *JAMA Oncology*.

eTable 1. RNAseq-Based Marker Nomination Criteria

Criterion	Cancer Criteria[a]	High-Grade Criteria	Alternate High-Grade Criteria
1	Gene length >500 bp	Gene length >500 bp	Gene length >500 bp
2	Differential expression (cancer versus normal) adjusted p-value <0.1	Differential expression adjusted p-value <0.1	Differential expression (low-grade versus high-grade cancers) adjusted p-value <0.1
3	log2 mean fold-change >0	$\log_2$ mean fold-change >0	$\log_2$ mean fold-change >0.5
4	>50% of cancer samples in the high-expressing samples	>85% of high-expressing samples in high-grade cancers via clustering statistics	>80% of high-expressing samples in high-grade cancers via clustering statistics
5	95th percentile normal expression <50 TPM	$\log_2$ fold-change (high-expressing sample mean versus low-expressing sample mean) >0	$\log_2$ mean expression of high-expressing samples >6
6	$\log_2$ fold-change (50th percentile cancer sample versus 75th percentile normal samples) >0	95th percentile normal expression <50 TPM	
7	$\log_2$ fold-change (90th percentile cancer sample versus 90th percentile normal samples) >1.9 or $\log_2$ fold-change (90th percentile cancer sample versus 50th percentile normal samples) >3.75	95th percentile high-grade expression >30 TPM	

Abbreviations: bp, base pairs; $\log_2$, log base 2; TPM, transcripts per million

[a] RNAseq-based analysis for identification of novel informative biomarkers utilized one set of criteria for cancer-associated genes (ie, expression in cancer significantly differs from expression in benign tissue) and two sets of criteria for high-grade cancer-associated genes (ie, expression in grade group 2 or greater cancer significantly differs from expression in grade group 1 cancer).

© 2024 Tosoian JJ et al. *JAMA Oncology*.

eTable 2. Indications for Prostate Biopsy in the External Validation Cohort

Indication for biopsy – No. (%)	Biopsy-Naïve (N=496)	Repeat Biopsy (N=247)	Total (N=743)
PSA >2.0 ng/mL	294 (59%)	141 (57%)	435 (59%)
Elevated PSA Velocity	95 (19%)	48 (19%)	143 (19%)
Abnormal DRE	94 (19%)	29 (12%)	123 (17%)
Prior ASAP or HGPIN	0 (0%)	19 (7.7%)	19 (2.6%)
Percent Free PSA <15%	9 (1.8%)	3 (1.2%)	12 (1.6%)
Other	4 (0.8%)	7 (2.8%)	11 (1.5%)

Abbreviations: ASAP, atypical small acinar proliferation; DRE, digital rectal exam; HGPIN, high-grade prostatic intraepithelial neoplasia; PSA, prostate-specific antigen.

Urine collection was performed per the identical NCI-EDRN protocol employed in the development cohort. The validation cohort underwent per-protocol blood collection as described.[1] Because all patients were enrolled with a clinical indication for post-PSA biomarker testing, we included the minority of patients with PSA values above 10 ng/mL (103, 14%). Additional eligibility criteria included availability of urine and clinical data for analysis. All patients underwent TRUS-guided systematic biopsy, of which 736 (99%) included ≥12 cores. Pathologic interpretation was performed by genitourinary pathologists at each academic center per the ISUP Consensus Conference.[2, 3] A randomized 10% of specimens were independently re-reviewed by central pathology. All participants provided informed consent.

© 2024 Tosoian JJ et al. *JAMA Oncology*.

eTable 3. Frequency of Inclusion and Cumulative Importance of the 17 Most Informative Markers Across 40 Elastic Net Models Assessed in Development

No.	Gene Name	Chromosome	Gene ID	Frequency	Cumulative Importance [a]
1	TMPRSS2-ERG	21-21	ENSG00000184012, ENSG00000157554	40	1265
2	SCHLAP1	2	ENSG00000281131	35	1582
3	OR51E2	11	ENSG00000167332	33	2006
4	APOC1	19	ENSG00000130208	31	456
5	PCAT14	22	ENSG00000280623	30	841
6	CAMKK2	12	ENSG00000110931	29	1604
7	PCA3	9	ENSG00000225937	28	1015
8	NKAIN1	1	ENSG00000084628	28	456
9	B3GNT6	11	ENSG00000198488	28	211
10	TFF3	21	ENSG00000160180	26	1329
11	SPON2	4	ENSG00000159674	26	1080
12	PCGEM1	2	ENSG00000227418	26	725
13	TRGV9	7	ENSG00000211695	24	955
14	TMSB15A	X	ENSG00000158164	22	548
15	ERG	21	ENSG00000157554	21	221
16	KLK4	19	ENSG00000167749	20	1094
17	HOXC6	12	ENSG00000197757	20	354

[a] Cumulative importance indicates the relative weight of marker importance summed across repeat samplings as derived by elastic net modeling.

© 2024 Tosoian JJ et al. *JAMA Oncology*.

eTable 4. Model Coefficients for the MPS2 and MPS2+ Models

Covariate	MPS2 [a]	MPS2+ [b]
(Intercept)	5.902430658	6.676363594
T2ERG	0.111906862	0.148584627
SCHLAP1	0.17335791	0.205268829
OR51E2	0.200676934	0.228791882
APOC1	-0.07916931	-0.08896388
PCAT14	0.14420976	0.16009867
CAMKK2	-0.26364401	-0.277941927
PCA3.1	0.080881661	0.074209893
NKAIN1	-0.06946207	-0.093791082
B3GNT6	0.047475092	0.072524885
TFF3	0.186395669	0.2128103
SPON2	0.156664808	0.1740959
PCGEM1	-0.16940833	-0.149084289
TRGV9	0.096184103	0.177972309
TMSB15A	0.151071453	0.214870771
ERG	0.023544761	0.030085251
KLK4	0.149451849	0.214609188
HOXC6	0.05612131	0
Age	0.000134221	0.021446485
African American	0.828856591	1.232493234
Family History	0.148709502	0.292757369
Abnormal DRE	0.888379309	1.094432439
Previous Biopsy	-0.8505938	-0.61694213
PSA	0.073709982	0.092554335
Prostate Volume	N/A	-0.024051593

[a] MPS2: Calibrated logit = -1.453526 + logit*1.302089
[b] MPS2+: Calibrated logit = -1.41207 + logit*1.077061

© 2024 Tosoian JJ et al. *JAMA Oncology*.

eTable 5. Characteristics of the NCI EDRN External Validation Population Stratified by Previous Biopsy Status

Characteristic	**Initial Biopsy (N=496)**	**Repeat Biopsy (N=247)**
Median age (IQR) – years	62 (56-67)	63 (59-68)
African-American [a] – No. (%)	70 (14%)	25 (10%)
Positive family history – No. (%)	134 (27%)	78 (32%)
Previous negative biopsy – No. (%)	0 (0%)	247 (100%)
Abnormal DRE – No. (%)	111 (22%)	28 (11%)
Median prostate volume [b] – mL	40 (29-51)	56 (39-81)
Median PSA (IQR) – ng/mL	5.0 (3.8-6.6)	7.2 (5.5-9.8)
Median PSA density [c] (IQR) – ng/mL^2	0.12 (0.08-0.19)	0.12 (0.09-0.20)
Median PHI (IQR)	40.8 (30.2-55.1)	39.3 (29.6-54.7)
Median PCA3 (IQR)	27.4 (12.4-61.1)	21.2 (10.0-45.1)
Median MPS (IQR)	34 (16-57)	35 (17-55)
Median MPS2 [d] (IQR)	0.20 (0.08-0.46)	0.06 (0.02-0.14)
Median MPS2+ [d] (IQR)	0.22 (0.08-0.53)	0.06 (0.02, 0.21)
Biopsy GG ≥2 – No. (%)	133 (27%)	18 (7.3%)

Abbreviations: DRE, digital rectal examination; GG, denotes grade group; IQR, interquartile range; MPS, MyProstateScore; MPS2, MyProstateScore 2.0; MPS2+, MyProstateScore 2.0 plus; PCA3, prostate cancer antigen 3; PHI, prostate health index; PSA, prostate-specific antigen

[a] African-American race was self-reported by participants. African-American race was assessed due to a well-established association with prostate cancer incidence, outcomes, and molecular prostate cancer subtypes.
[b] Measured by transrectal ultrasound.
[c] PSA density equals serum PSA divided by prostate volume.
[d] MPS2 and MPS2+ values are reported on a continuous scale as the likelihood of detecting high-grade prostate cancer (ie, grade group 2 or greater) on biopsy.

© 2024 Tosoian JJ et al. *JAMA Oncology*.

eTable 6. Performance Measure Calculations for High-Grade Cancer using PSA Alone, Prostate Cancer Prevention Trial Risk Calculator, Prostate Health Index (PHI), Derived Multiplex 2-Gene and 3-Gene Models, MyProstateScore (MPS), MPS2, and MPS2 Plus Prostate Volume (MPS2+) in the EDRN Validation Cohort

	Sensitivity	Specificity[a]	NPV	PPV
**Overall (n = 743)**				
PSA	95%	64/592 (11%)	64/71 (90%)	144/672 (21%)
Prostate Cancer Prevention Trial risk calculator	95%	117/592 (20%)	117/124 (94%)	144/619 (23%)
PHI	95%	153/592 (26%)	153/160 (96%)	144/583 (25%)
Derived multiplex 2-gene model	95%	160/592 (27%)	160/167 (96%)	144/576 (25%)
Derived multiplex 3-gene model	95%	101/592 (17%)	101/108 (94%)	144/635 (23%)
MPS	95%	136/592 (23%)	136/144 (94%)	143/599 (24%)
MPS2	95%	219/592 (37%)	219/227 (97%)	143/516 (28%)
MPS2+	95%	240/592 (41%)	240/248 (97%)	143/495 (29%)
**Initial biopsy (n = 496)**				
PSA	95%	55/363 (15%)	55/62 (89%)	126/434 (29%)
Prostate Cancer Prevention Trial risk calculator	95%	97/363 (27%)	97/103 (94%)	127/393 (32%)
PHI	95%	107/363 (30%)	107/113 (95%)	127/383 (33%)
Derived multiplex 2-gene model	95%	110/363 (30%)	110/116 (95%)	127/380 (33%)
Derived multiplex 3-gene model	95%	61/363 (17%)	61/67 (91%)	127/429 (30%)
MPS	95%	98/363 (27%)	98/105 (93%)	126/391 (32%)
MPS2	95%	126/363 (35%)	126/133 (95%)	126/363 (35%)
MPS2+	95%	152/363 (42%)	152/159 (96%)	126/337 (37%)
**Repeat biopsy (n = 247)**				
PSA	94.4%	34/229 (15%)	34/35 (97%)	17/212 (8.0%)
Prostate Cancer Prevention Trial risk calculator	94.4%	48/229 (21%)	48/49 (98%)	17/198 (8.6%)
PHI	94.4%	20/229 (8.7%)	20/21 (95%)	17/226 (7.5%)
Derived multiplex 2-gene model	94.4%	33/229 (14%)	33/34 (97%)	17/213 (8.0%)
Derived multiplex 3-gene model	94.4%	37/229 (16%)	37/38 (97%)	17/209 (8.1%)
MPS	94.4%	34/229 (15%)	34/36 (97%)	17/212 (8.0%)
MPS2	94.4%	106/229 (46%)	106/107 (99%)	17/140 (12%)
MPS2+	94.4%	117/229 (51%)	117/118 (99%)	17/129 (13%)

Abbreviations: NPV, negative predictive value; PPV, positive predictive value.
[a]Clinically, specificity represents the proportion of unnecessary biopsies avoided with use of the test at the 95% sensitivity threshold to select patients for biopsy.

© 2024 Tosoian JJ et al. *JAMA Oncology*.

eTable 7. Clinical Performance of High Sensitivity MPS2 Threshold Values for High-Grade Cancer in the Initial Biopsy Subpopulation of the External Validation Population

Threshold	Sensitivity	Specificity	NPV	PPV
MPS2+				
0.05	97%	21%	95%	31%
0.06	97%	25%	96%	32%
0.07	96%	29%	95%	33%
0.075	96%	31%	96%	34%
0.08	95%	32%	95%	34%
0.09	95%	35%	95%	35%
0.10	95%	38%	95%	36%
0.11	95%	42%	96%	37%
0.12	92%	44%	94%	38%
0.13	92%	48%	95%	39%
0.14	92%	51%	94%	41%
0.15	90%	53%	94%	41%
0.175	88%	57%	93%	43%
0.20	86%	60%	92%	44%
MPS2				
0.05	96%	21%	94%	31%
0.06	96%	25%	95%	32%
0.07	96%	28%	95%	33%
0.075	96%	31%	96%	34%
0.08	95%	33%	94%	34%
0.087	95%	35%	95%	35%
0.09	94%	36%	94%	35%
0.10	94%	39%	95%	36%
0.11	93%	41%	94%	37%
0.12	92%	46%	94%	38%
0.13	91%	49%	94%	39%
0.14	89%	52%	93%	41%
0.15	89%	54%	93%	41%
0.175	86%	59%	92%	44%
0.20	83%	63%	91%	45%
Biomarkers Only				
0.05	95%	20%	92%	30%
0.06	95%	28%	94%	33%
0.07	95%	33%	94%	34%
0.075	95%	35%	95%	35%
0.077	95%	35%	95%	35%
0.08	94%	37%	94%	35%
0.09	92%	41%	93%	36%
0.10	90%	44%	92%	37%

© 2024 Tosoian JJ et al. *JAMA Oncology*.

0.11	88%	46%	92%	38%
0.12	85%	50%	90%	38%
0.15	75%	59%	87%	40%
0.175	72%	66%	86%	44%
0.20	68%	71%	86%	46%

© 2024 Tosoian JJ et al. *JAMA Oncology*.

eTable 8. Clinical Performance of High Sensitivity MPS2 Threshold Values for High-Grade Cancer in the Repeat Biopsy Subpopulation of the External Validation Population

Threshold	Sensitivity	Specificity	NPV	PPV
MPS2+				
0.04	100%	40%	100%	12%
0.05	94%	47%	99%	12%
0.058	94%	51%	99%	13%
0.06	88%	52%	98%	13%
0.07	83%	57%	98%	13%
0.08	78%	59%	97%	13%
0.09	67%	62%	96%	12%
0.10	67%	62%	96%	12%
MPS2				
0.04	94%	42%	99%	11%
0.044	94%	46%	99%	12%
0.05	89%	48%	98%	12%
0.058	89%	51%	98%	12%
0.06	89%	52%	98%	13%
0.07	72%	57%	96%	12%
0.08	67%	62%	96%	12%
0.09	67%	66%	96%	13%
0.10	67%	68%	96%	14%
Biomarkers Only				
0.04	100%	22%	100%	9.1%
0.05	100%	26%	100%	9.6%
0.06	100%	32%	100%	10%
0.07	100%	35%	100%	11%
0.08	94%	42%	99%	11%
0.09	83%	45%	97%	11%
0.10	78%	49%	97%	11%

© 2024 Tosoian JJ et al. *JAMA Oncology*.

eTable 9. Characteristics of the Secondary Development Cohort for Patients Undergoing Initial Biopsy at U-M

Characteristic	**Initial Biopsy (N=598)**
Median age (IQR) – years	63 (57-58)
African-American [a] – No. (%)	24 (4.0%)
Positive family history – No. (%)	157 (26%)
Previous negative biopsy – No. (%)	0 (0%)
Abnormal DRE – No. (%)	87 (15%)
Median prostate volume [b] – mL	36 (35-61)
Median PSA (IQR) – ng/mL	5.4 (4.5-6.8)
Median PSA density [c] (IQR) – ng/mL^2	0.12 (0.08-0.16)
Median MPS2 [d] (IQR)	0.20 (0.08-0.46)
Median MPS2+ [d] (IQR)	0.22 (0.08-0.53)
Biopsy GG ≥2 – No. (%)	133 (27%)

[a] African-American race was self-reported by participants. African-American race was assessed due to a well-established association with prostate cancer incidence, outcomes, and molecular prostate cancer subtypes.
[b] Measured by transrectal ultrasound.
[c] PSA density equals serum PSA divided by prostate volume.
[d] MPS2 and MPS2+ values are reported on a continuous scale as the likelihood of detecting high-grade prostate cancer on biopsy.

© 2024 Tosoian JJ et al. *JAMA Oncology*.

eTable 10. Clinical Performance of Secondary Initial Biopsy (iMPS2) Models for High-Grade Cancer in the Initial Biopsy Subpopulation of the External Validation Cohort (n = 496)

Threshold	Sensitivity	Specificity	NPV	PPV
iMPS2+				
0.05	99%	14%	96%	30%
0.095	96%	31%	95%	34%
0.115	96%	36%	96%	35%
0.135	95%	43%	96%	38%
0.145	93%	46%	95%	38%
0.16	92%	48%	94%	40%
0.19	92%	53%	95%	42%
0.20	90%	55%	94%	42%
0.25	86%	63%	92%	46%
iMPS2				
0.05	97%	18%	94%	30%
0.095	96%	33%	95%	34%
0.115	95%	39%	95%	36%
0.135	93%	46%	95%	39%
0.145	92%	48%	94%	39%
0.16	90%	53%	94%	41%
0.19	87%	56%	92%	42%
0.20	86%	59%	92%	43%
0.25	80%	65%	90%	46%
Biomarkers Only				
0.05	96%	15%	92%	29%
0.095	93%	35%	93%	34%
0.115	91%	40%	92%	36%
0.135	90%	46%	92%	38%
0.145	90%	49%	93%	39%
0.16	86%	53%	91%	40%
0.19	80%	60%	90%	43%
0.20	77%	63%	88%	44%
0.25	72%	69%	87%	46%

© 2024 Tosoian JJ et al. *JAMA Oncology*.

eTable 11. Model Coefficients from MPS2 Redevelopment Using Maximum Grade Group From All Biopsy and Surgical Specimens

Covariate	MPS2	RP-Derived MPS2[a]	MPS2+	RP-Derived MPS2+[a]
(Intercept)	5.902430658	5.36963703	6.676363594	5.66713268
T2ERG	0.111906862	0.12028164	0.148584627	0.15333868
SCHLAP1	0.17335791	0.15765111	0.205268829	0.14709972
OR51E2	0.200676934	0.17998234	0.228791882	0.15904006
APOC1	-0.07916931	-0.0798262	-0.08896388	-0.0924547
PCAT14	0.14420976	0.05653337	0.16009867	0.08193915
CAMKK2	-0.26364401	-0.2662075	-0.277941927	-0.2934813
PCA3.1	0.080881661	0.12143166	0.074209893	0.09433227
NKAIN1	-0.06946207	N/A	-0.093791082	N/A
B3GNT6	0.047475092	0.04017777	0.072524885	0.05564982
TFF3	0.186395669	0.20852164	0.2128103	0.24750486
SPON2	0.156664808	0.08542159	0.1740959	0.03934708
PCGEM1	-0.16940833	-0.1154365	-0.149084289	-0.1369629
TRGV9	0.096184103	0.2130775	0.177972309	0.24262976
TMSB15A	0.151071453	N/A	0.214870771	N/A
ERG	0.023544761	N/A	0.030085251	N/A
KLK4	0.149451849	N/A	0.214609188	N/A
HOXC6	0.05612131	0.07807035	0	0.06422307
ACSM1	N/A	0	N/A	-5.73E-05
LRRN1	N/A	-0.0212629	N/A	0
MS4A8	N/A	0.00093909	N/A	0.02808881
GRIN3A	N/A	0	N/A	-0.0040861
Age	0.000134221	0.00168473	0.021446485	0.0210196
African American	0.828856591	0.47329332	1.232493234	0.56924532
Family History	0.148709502	0.00459593	0.292757369	0.0820011
Abnormal DRE	0.888379309	0.68733551	1.094432439	0.86237867
Previous Biopsy	-0.8505938	-0.6063101	-0.61694213	-0.4762385
PSA	0.073709982	0.03565447	0.092554335	0.10335382
Prostate Volume	N/A	N/A	-0.024051593	-0.0255555

Abbreviations: DRE, digital rectal exam; MPS2, MyProstateScore2.0; MPS2+ MyProstateScore 2.0 plus; PSA, prostate-specific antigen; RP radical prostatectomy and/or repeat biopsy.

[a] Considering potential misclassification of high-grade prostate cancer due to biopsy undersampling, we evaluated MPS2 models including pathologic data obtained subsequent to study urine collection (e.g., repeated biopsy, radical prostatectomy). Of 761 patients in the development set, 382 (50%) underwent additional biopsy (N=201) and/or radical prostatectomy (N=217), and 71 (11%) were upgraded to high-grade prostate cancer. The table includes parameters from models derived based on the highest cancer grade detected (i.e., RP-derived models). Of the 17 informative markers in the MPS2 model, 13 were retained in the RP-derived model. The direction of biomarker association with the outcome was unchanged for all markers. The AUC of the cross validated models differed by 1% for MPS2 (0.802 vs. 0.792) and 0.1% for MPS2+ (0.821 vs. 0.822).

© 2024 Tosoian JJ et al. *JAMA Oncology*.

eFigure 1. Heatmap Illustrating Differential Expression of RNAseq Target Genes

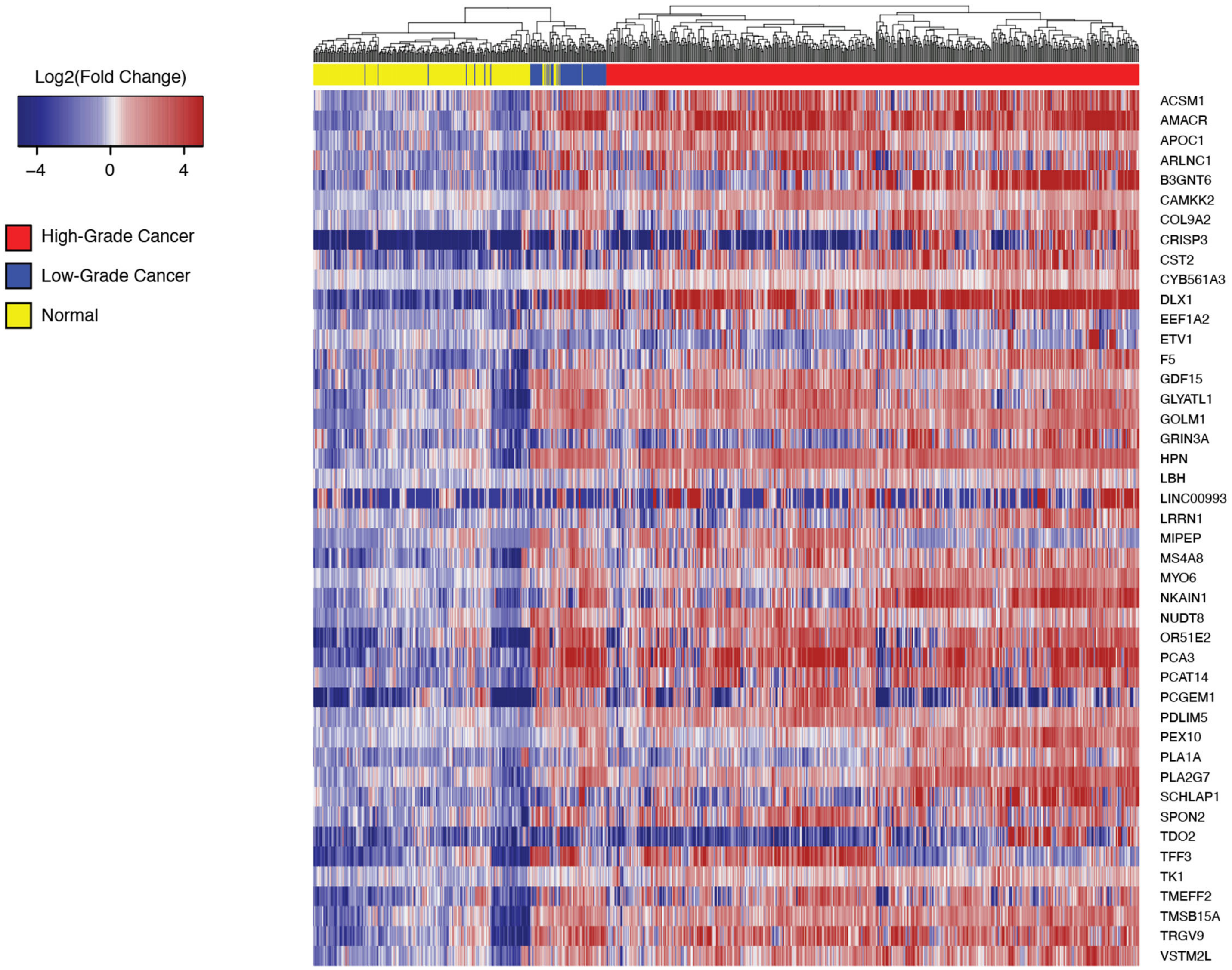

Legend: Shown is a heatmap illustrating differential expression of 44 target genes in the discovery set as determined by RNAseq. The color bar below the column-wise hierarchical clustering tree represents sample pathology. Normal samples, defined as those with no cancer, are shown in yellow (N=220), low-grade cancers in blue (N=71), and higher-grade cancers (grade group 2 or higher) in red (N=484). Red, white, and blue cells in rows represent differential gene expression, calculated as $\log_2$(fold change in gene expression) divided by mean expression in benign samples. Blue represents decreased expression relative to normal, red represents increased expression relative to normal, and white represents no difference. The column-wise dendrogram represents hierarchical clustering among high-grade and low-grade cancer to normal samples.

© 2024 Tosoian JJ et al. *JAMA Oncology*.

eFigure 2. Box and Dot Plots of Gene Expression in Benign, Low-Grade Cancer, and High-Grade Cancer Tissue in the Discovery Set

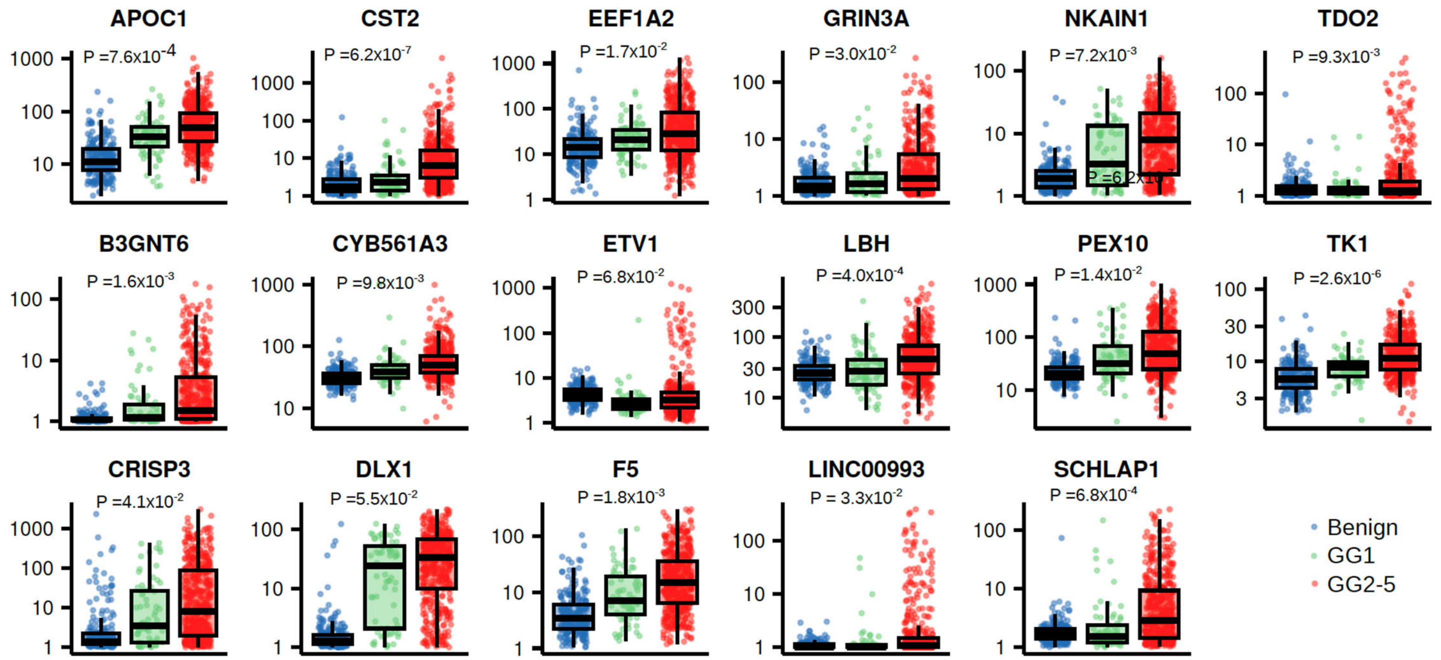

Legend: Shown are box and dot plots of gene expression from tissue-based RNAseq analyses for the 17 candidate biomarkers meeting pre-defined nomination criteria for high-grade prostate cancer. Plots illustrate log-transformed RNAseq-derived transcripts per million (TPM) for benign prostate tissue (blue), GG1 cancer (green), and GG≥2 cancer (red). Differential expression log fold-change values for all genes are included in eTable 5.

© 2024 Tosoian JJ et al. *JAMA Oncology*.

eFigure 3. Box and Dot Plots of Gene Expression in Benign vs Prostate Cancer Tissue in the Discovery Set

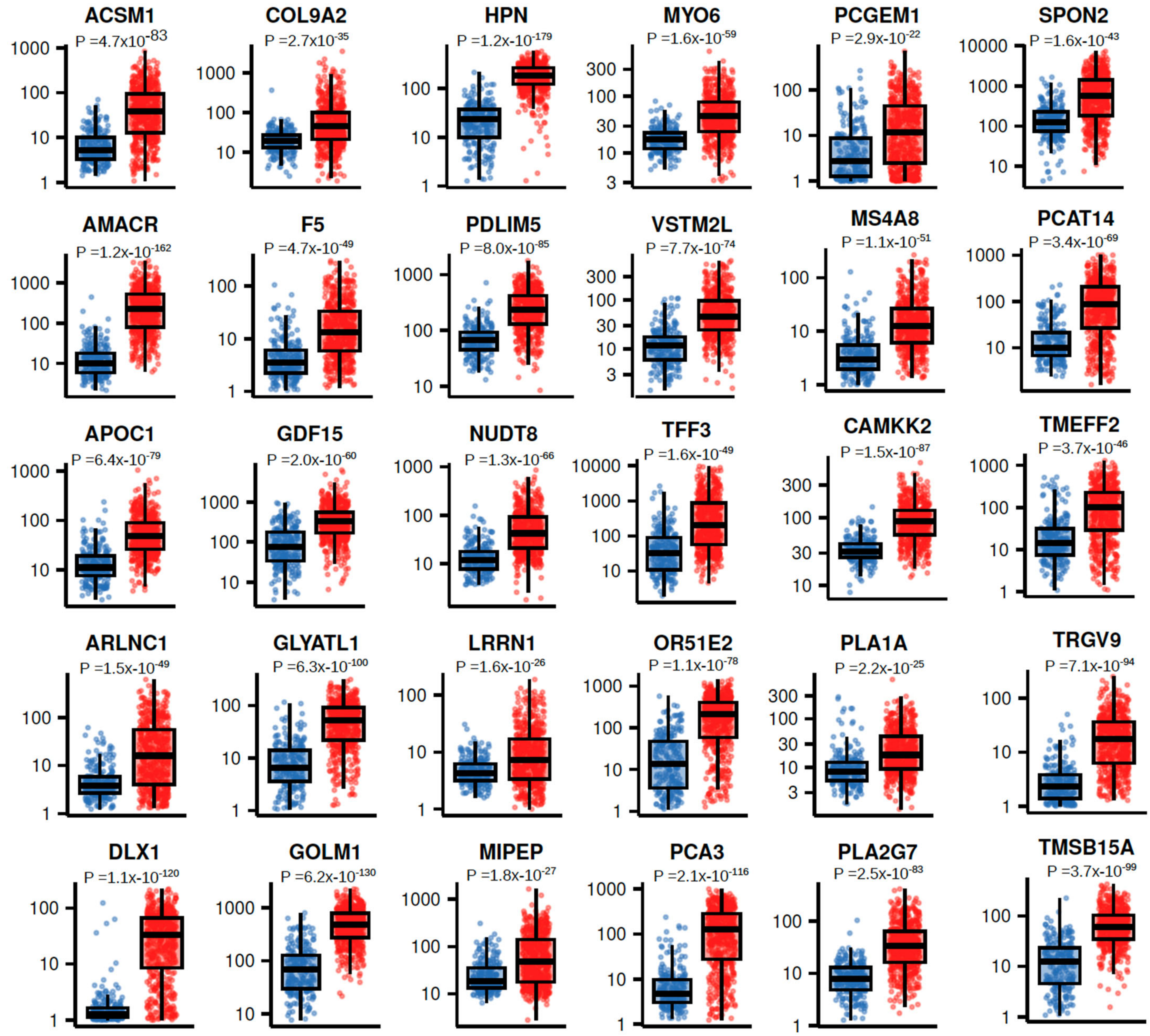

Legend: Shown are box and dot plots of gene expression from tissue-based RNAseq analyses for the 30 candidate biomarkers meeting pre-defined nomination criteria for prostate cancer. Plots illustrate log-transformed RNAseq-derived transcripts per million (TPM) for benign prostate tissue (blue) and prostate cancer (red). Differential expression log fold-change values for all genes are included in eTable 5.

© 2024 Tosoian JJ et al. *JAMA Oncology*.

eFigure 4. Flow Diagram of the University of Michigan Development Cohort

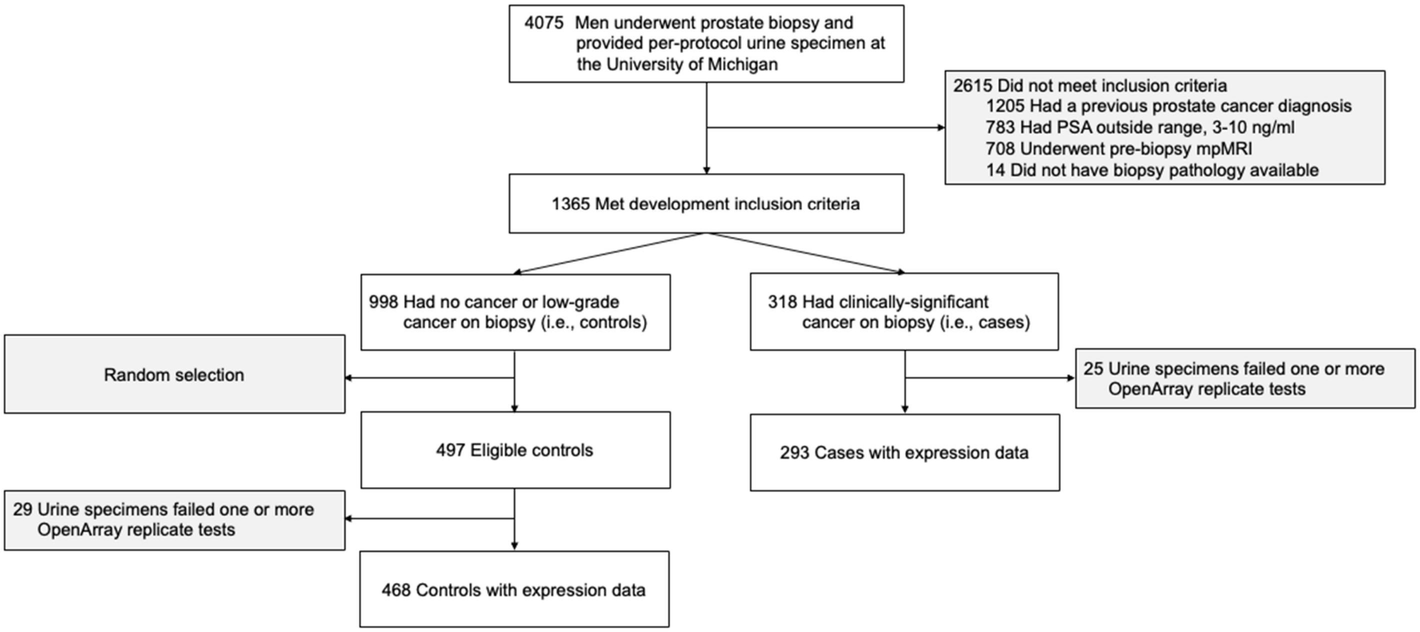

Abbreviations: mpMRI, multi-parametric magnetic resonance imaging; PSA, prostate-specific antigen,

Legend: Among 1365 eligible subjects in the University of Michigan biospecimen database (IRB HUM00042749), 318 were found to have high-grade cancer on biopsy (i.e., cases). Power analysis demonstrated a 1:1 case to control (i.e., no cancer or grade group 1 cancer on biopsy) design with N=300 per group would provide >95% power to detect differential expression of up to 20 candidate biomarkers.[4, 5] Considering the incremental power gained from additional controls, we assessed 497 eligible controls with adequate urine for study inclusion.

OpenArray™ technology (Thermo Fisher Scientific, Waltham, MA, USA) is a high-throughput real-time quantitative polymerase chain reaction (qPCR) method that allows for rapid screening of multiple TaqMan™ assays across samples. This method uses an array of 3072 through-holes run on the QuantStudio 12K Flex Real-Time PCR System with an OpenArray™ block. RNA isolation for the 54-gene OpenArray™ panel was performed using the MagMAX™ mirVana™ Total RNA Isolation Kit. Briefly, 500 microliters of a 1 to 1 mixture of urine and Hologic transport media were mixed 1 to 1 with Lysis Binding Mix. Binding Beads Mix was added to enrich nucleic acids, followed by TURBO DNase digestion and RNA elution. For high-throughput RNA extraction, urine samples were processed using the semi-automatic KingFisher Flex System (Thermo Fisher Scientific). All samples were run in triplicate.

After RNA extraction, 16 microliters of RNA were used to synthesize cDNA with SuperScript™ IV VILO™ Master Mix, followed by pre-amplification with TaqMan™ PreAmp Master Mix (Thermo Fisher Scientific). For each sample, 2.5 microliters of pre-amplified cDNA and 2.5 microliters of 2× TaqMan OpenArray™ Master Mix were loaded into 384-well plates per manufacturer instructions. The QuantStudio 12K Flex OpenArray™ AccuFill System transferred the mix to the TaqMan OpenArray™ plate. Amplification was performed using the QuantStudio™ 12K Flex Real Time PCR System, and the delta-delta cycle threshold method was used for analysis with the QuantStudio™ 12K Flex Software. In the development cohort, the OpenArray™ assay failed one or more replicates in 29 eligible controls and 25 eligible cases, yielding the final cohort for analysis.

The measure of gene expression was the cycle threshold (Ct), defined as the number of amplification cycles required for fluorescence to exceed background level. Ct values are inversely related to the quantity of nucleic acid in a sample, with lower Ct values reflecting increased expression of the target gene. All samples were run in triplicate, and mean Ct values of the 54 candidate markers were normalized to the housekeeping gene *KLK3* using the formula $-[Ct_{target} - Ct_{KLK3}]$. The normalized mean Ct was used for model building.

© 2024 Tosoian JJ et al. *JAMA Oncology*.

eFigure 5. Postcalibration Curves of MPS2 and MPS2+ in the Development Cohort

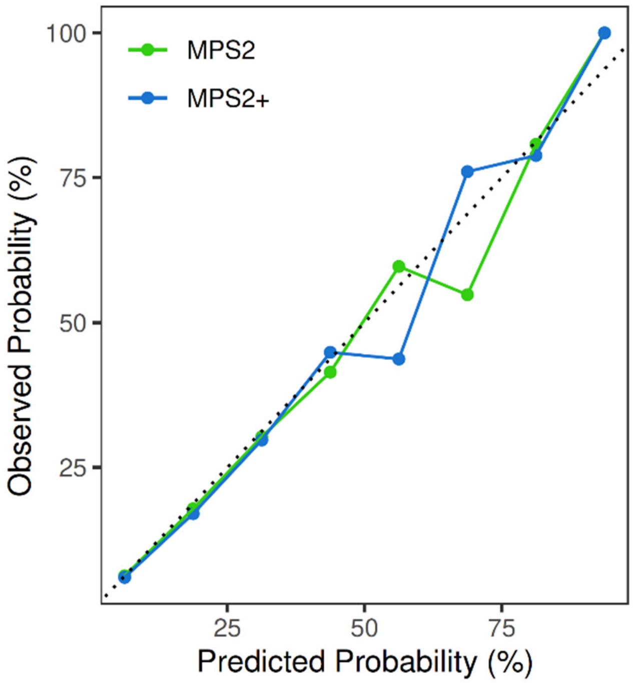

Legend: Model calibration reflects agreement between the predicted outcomes from the model and observed outcomes. The MPS2 models were calibrated to account for differences in outcome prevalence between the development and validation cohorts.[6] Two calibration methods[7, 8] were applied to a re-sampled development set with outcome prevalence matched to the validation cohort: i) recalibration in the large, which includes re-estimation of the model intercept, and ii) logistic recalibration, which includes re-estimation of model intercept and slope.[8] The latter method provided superior performance and was used for calibration. The calibrated model was locked, and internal cross validation was performed using the *train* function from the R package *caret*.[9] Calibration was reported graphically as observed versus predicted risk of outcome.[6] Shown are post-calibration curves for MPS2 (green) and MPS2+ (blue) in the development set re-sampled to match high-grade cancer prevalence in the validation cohort. The observed prevalence of GG≥2 cancer closely approximates the MPS2 and MPS2+ predicted probabilities, reflecting good calibration.

© 2024 Tosoian JJ et al. *JAMA Oncology*.

eFigure 6. MPS2 and MPS2+ Cross-Validated Area Under the ROC Curves for High-Grade Prostate Cancer in the Development Cohort

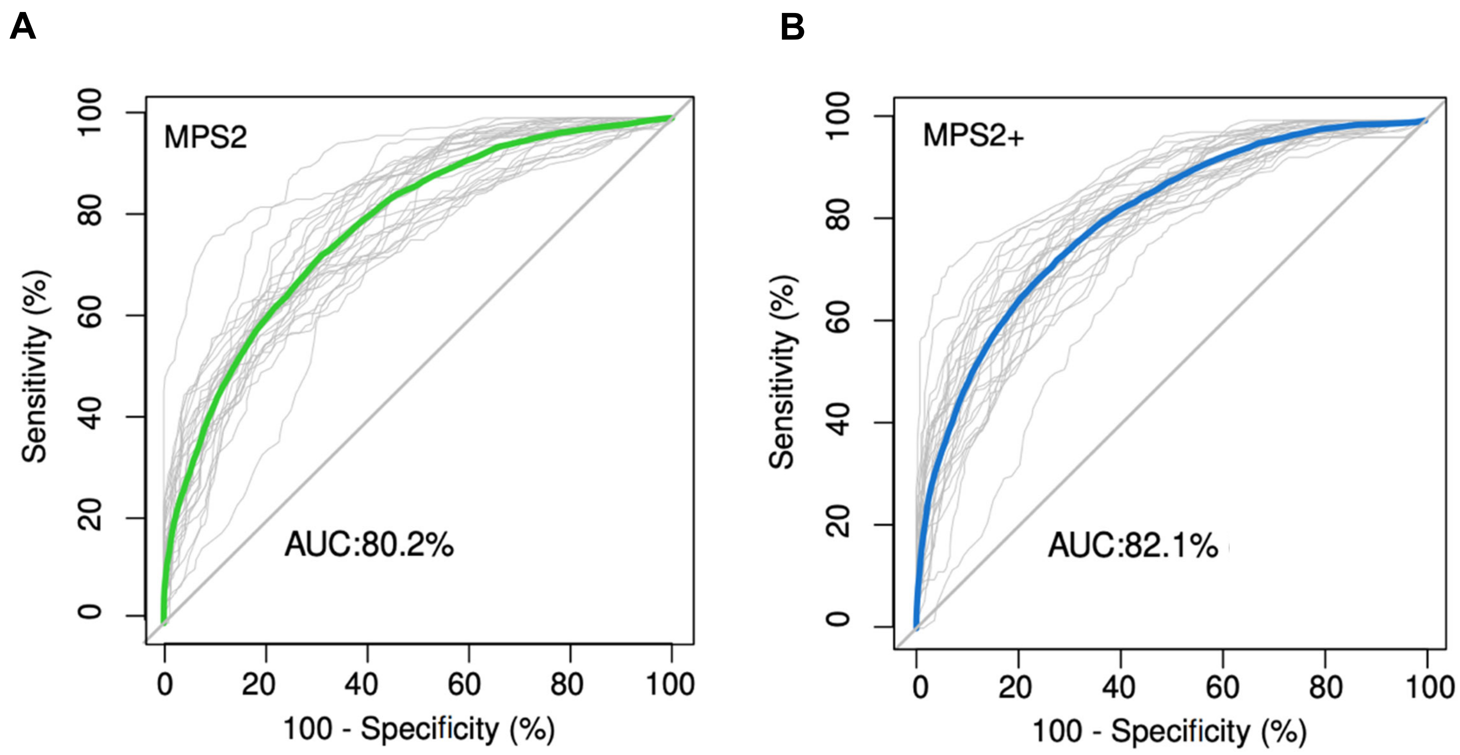

Legend: Receiver-operating characteristic (ROC) curves and the corresponding areas under the curve (AUC) for the cross-validated MPS2 (Panel A) and MPS2+ (Panel B) tests. The panels include ROC curves of individual cross-validation folds (thin gray lines) and the mean ROC of all cross-validation folds for MPS2 (green) and MPS2+ (blue).

© 2024 Tosoian JJ et al. *JAMA Oncology*.

eFigure 7. Flow Diagram of the NCI EDRN External Validation Cohort

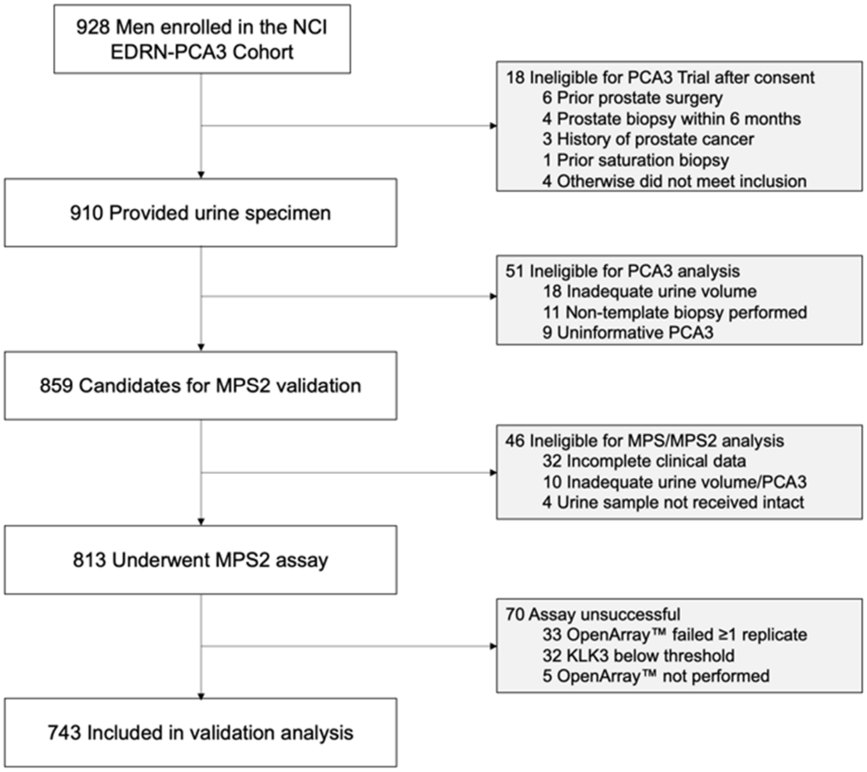

Legend: Shown is the external validation cohort comprised of men undergoing prostate biopsy in the National Cancer Institute – Early Detection Research Network (NCI-EDRN) PCA3 Trial. Of 859 men participating in the PCA3 trial, 46 (5.4%) were ineligible for the current analysis due to inadequate urine volume or unavailable clinical data. Median days from urine collection to biopsy was 0 (IQR 0-0). Of 813 eligible participants, the MPS2 assay was successfully performed in 743 (91%), yielding the final external validation population. The PCA3 Validation Trial protocol is accessible at: https://edrn.nci.nih.gov/data-and-resources/specimen-reference-sets/prostate-reference-sets/

© 2024 Tosoian JJ et al. *JAMA Oncology*.

eFigure 8. MPS2 Values by Biopsy Pathology in the External Validation Cohort

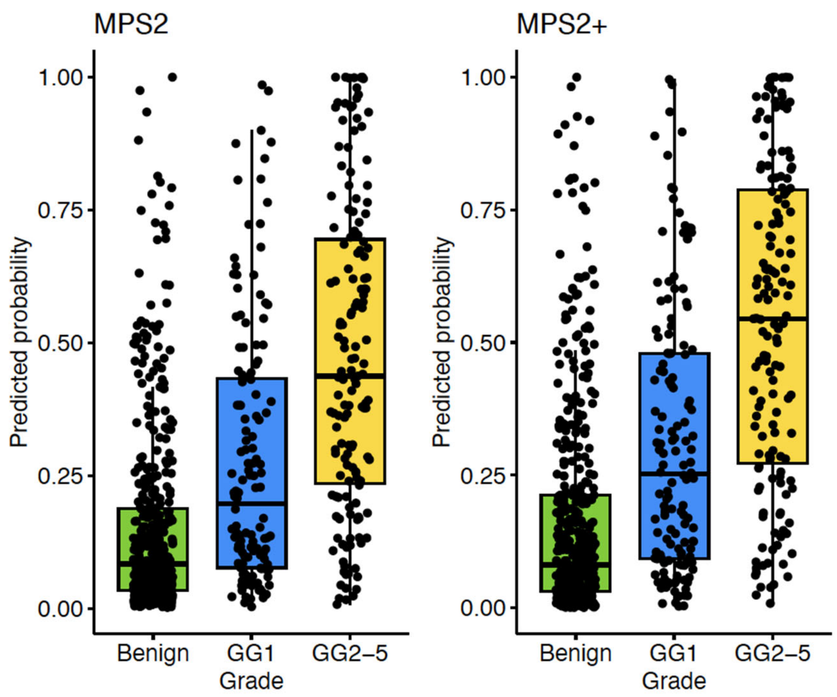

Legend: Box and dot plots illustrating the distribution of MPS2 and MPS2+ values in men a negative biopsy (green), GG1 cancer on biopsy (blue), and GG≥2 cancer on biopsy (yellow) in the external validation cohort. P-values were ≤0.001 for pairwise comparisons of GG≥2 cancer with negative biopsy and GG1 cancer for both MPS2 models.

© 2024 Tosoian JJ et al. *JAMA Oncology*.

eFigure 9. MPS2 and MPS2+ Area Under the ROC Curves for High-Grade Prostate Cancer in the External Validation Cohort

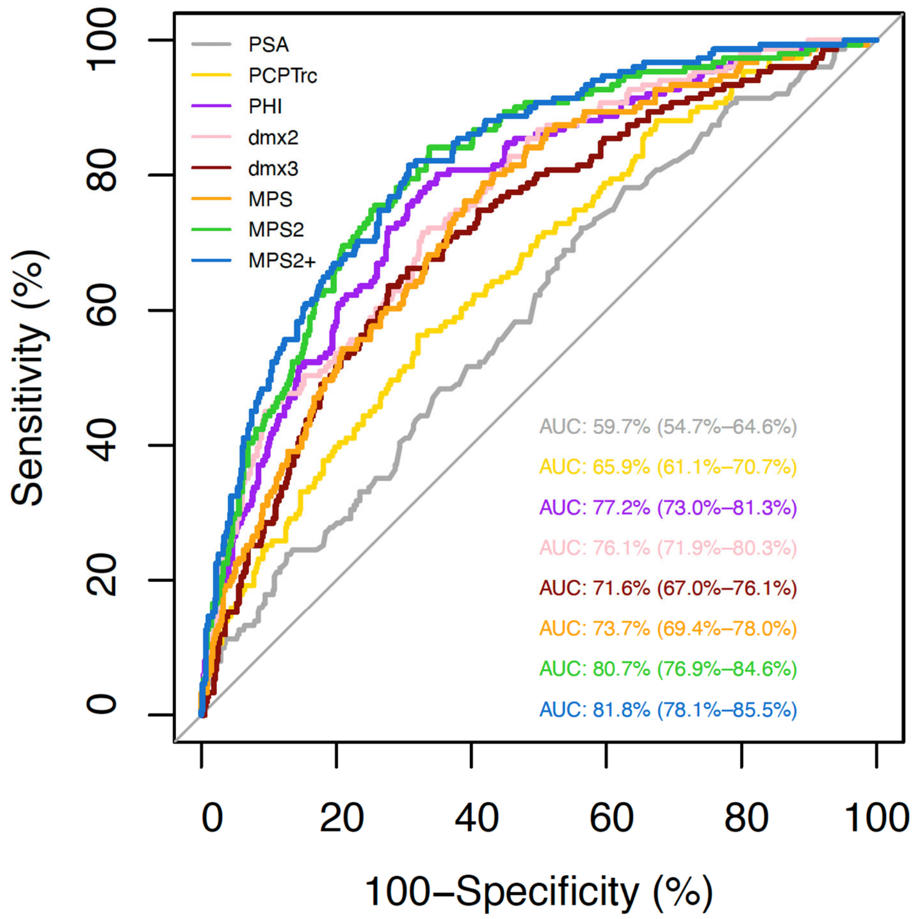

Legend: Receiver-operating characteristic curves and areas under the curve (AUC) for PSA (gray), PCPTrc (yellow), PHI (purple), dmx2 (pink), dmx3 (maroon), MPS (orange), MPS2 (green), and MPS2+ (blue) in the external validation cohort.

© 2024 Tosoian JJ et al. *JAMA Oncology*.

eFigure 10. Postcalibration Curves of iMPS2 and iMPS2+ in the External Validation Cohort

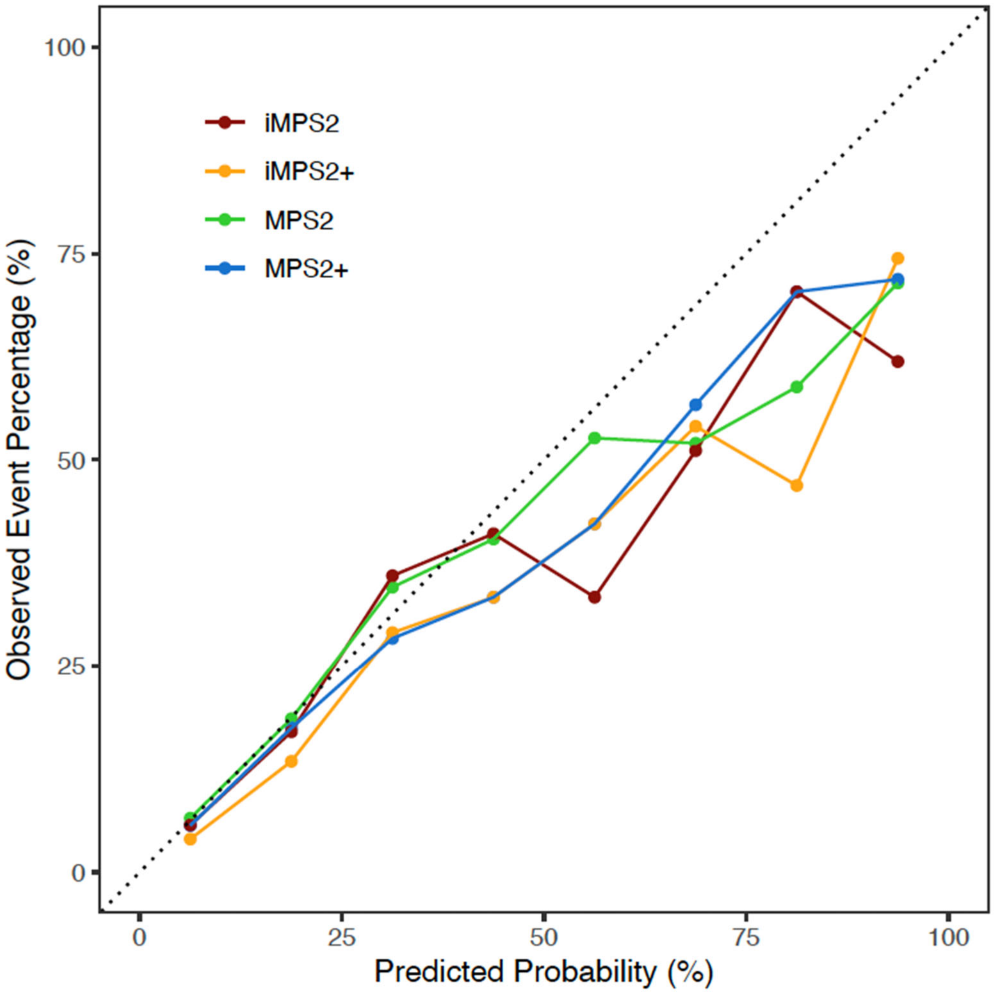

Legend: Calibration curves for high-grade prostate cancer for alternative initial biopsy models iMPS2 (maroon) and iMPS+ (orange) plotted with the MPS2 (green) and MPS2+ (blue) models.

© 2024 Tosoian JJ et al. *JAMA Oncology*.

eReferences.

1. Wei JT, Feng Z, Partin AW, et al. Can urinary PCA3 supplement PSA in the early detection of prostate cancer? *J Clin Oncol*. Dec 20 2014;32(36):4066-72. doi:10.1200/JCO.2013.52.8505
2. Epstein JI, Allsbrook WC, Jr., Amin MB, Egevad LL, Committee IG. The 2005 International Society of Urological Pathology (ISUP) Consensus Conference on Gleason Grading of Prostatic Carcinoma. *Am J Surg Pathol*. Sep 2005;29(9):1228-42. doi:10.1097/01.pas.0000173646.99337.b1
3. Epstein JI, Egevad L, Amin MB, et al. The 2014 International Society of Urological Pathology (ISUP) Consensus Conference on Gleason Grading of Prostatic Carcinoma: Definition of Grading Patterns and Proposal for a New Grading System. *Am J Surg Pathol*. Feb 2016;40(2):244-52. doi:10.1097/PAS.0000000000000530
4. Ching T, Huang S, Garmire LX. Power analysis and sample size estimation for RNA-Seq differential expression. *RNA*. Nov 2014;20(11):1684-96. doi:10.1261/rna.046011.114
5. Wang Z, Luo X, Chang YC. Assessing the predictive power of newly added biomarkers. *Biom J*. Sep 2015;57(5):797-807. doi:10.1002/bimj.201400210
6. Moons KG, Altman DG, Reitsma JB, et al. Transparent Reporting of a multivariable prediction model for Individual Prognosis or Diagnosis (TRIPOD): explanation and elaboration. *Ann Intern Med*. Jan 6 2015;162(1):W1-73. doi:10.7326/m14-0698
7. Thompson IM, Ankerst DP, Chi C, et al. Assessing prostate cancer risk: results from the Prostate Cancer Prevention Trial. *J Natl Cancer Inst*. Apr 19 2006;98(8):529-34. doi:10.1093/jnci/djj131
8. Vergouwe Y, Nieboer D, Oostenbrink R, et al. A closed testing procedure to select an appropriate method for updating prediction models. *Stat Med*. Dec 10 2017;36(28):4529-4539. doi:10.1002/sim.7179
9. Kuhn M. Building Predictive Models in R Using the caret Package. *Journal of Statistical Software*. 11/10 2008;28(5):1 - 26. doi:10.18637/jss.v028.i05

© 2024 Tosoian JJ et al. *JAMA Oncology*.
