# Supplementary material for: Development and Validation of an 18-Gene Urine Test for High-Grade Prostate Cancer
Source: JAMA Oncol. 2024 Apr 18;10(6):726–36. doi: 10.1001/jamaoncol.2024.0455 (PMC11190811; doi:10.1001/jamaoncol.2024.0455)
Supplement: Supplement 4. — Group Information. EDRN-PCA3 Study Group [file jamaoncol-e240455-s004.pdf]

Supplemental Online Content: Nonauthor Collaborators
*First name, last name, and suffix (if applicable) are required and will appear in PubMed.

***Group Name(s): EDRN-PCA3 Study Group**

***First Name and Middle Initial(s)**	***Last Name**	***Suffix (eg, Jr, III)**	Academic Degrees	Institution	Location (city, state/province, country)	Role or Contribution, eg, chair, principal investigator	Group (if more than 1 Group listed in the byline) and/or Subgroup (eg, Steering Committee)
Ian M.	Thompson	Jr	MD	University of Texas Health Sciences Center	San Antionio, Texas	Member	
Mohamed	Bidair		MD	San Diego Clinical Trials	San Diego, Texas	Member	
Adam	Kibel		MD	Brigham and Women's Hospital	Boston, Massachusetts	Member	
Daniel W.	Lin		MD	University of Washington Medical Center	Seattle, Washington	Member	
Yair	Lotan		MD	University of Texas Southwestern Medical Center	Dallas, Texas	Member	
Alan	Partin		MD, PhD	Johns Hopkins Hospital	Baltimore, Maryland	Member	
Samir	Taneja		MD	New York University School of Medicine	New York, New York	Member	
David H.	Howard		PhD	Department of Biostatistics, Rollins School of Public Health, Emory University	Atlanta, Georgia	Member	
Meredith M.	Regan		DSci	Department of Biostatistics and Computational Biology, Dana-Farber Cancer Institute, Harvard Medical School	Boston, Massachusetts	Member	
Jack	Groskopf		PhD	Hologic Inc	San Diego, California	Member	
Jonathan	Chipman		MS	Department of Biostatistics and Comutational Biology, Dana-Farber Cancer Institute, Harvard Medical School	Boston, Massachusetts	Member	
Dattatraya H.	Patil		MBBS, MPH	Department of Urology, Emory University School of Medicine	Atlanta, Georgia	Member	
Douglas S.	Scherr		MD	Department of Urology, Weill-Cornell Medical Center	New York, New York	Member	
Jacob	Kagan		PhD	Division of Cancer Prevention, National Cancer Institute	Bethesda, Maryland	Member	
Jing	Fan		MS	Luneenfeld-Tanenbaum Research Institute, Mount Sinai Hospital	Toronto, California	Member	

Supplemental Online Content: Nonauthor Collaborators
*First name, last name, and suffix (if applicable) are required and will appear in PubMed.

***First Name and Middle Initial(s)**	***Last Name**	***Suffix (eg, Jr, III)**	Academic Degrees	Institution	Location (city, state/province, country)	Role or Contribution, eg, chair, principal investigator	Group (if more than 1 Group listed in the byline) and/or Subgroup (eg, Steering Committee)
Aron Y.	Joon		MS	Department of Biostatistics, The University of Texas, MD Anderson Cancer Center	Houston, Texas	Member	
Leonidas E.	Bantis		PhD	Department of Biostatistics, The University of Texas, MD Anderson Cancer Center	Houston, Texas	Member	
Mark A.	Rubin		MD	Department of Pathology, Weill-Cornell Medical Center	New York, New York	Member	
