# Supplementary material for: Development and Validation of an 18-Gene Urine Test for High-Grade Prostate Cancer
Source: JAMA Oncol. 2024 Apr 18;10(6):726–36. doi: 10.1001/jamaoncol.2024.0455 (PMC11190811; doi:10.1001/jamaoncol.2024.0455)
Supplement: Supplement 5. — Data Sharing Statement [file jamaoncol-e240455-s005.pdf]

Data Sharing Statement

Tosoian. Development and Validation of an 18-Gene Urine Test for High-Grade Prostate Cancer. *JAMA Oncol*. Published April 18, 2024. doi:10.1001/jamaoncol.2024.0455

Data
Data available: No

Additional Information
Explanation for why data not available: As the data represented in this publication are from a blinded NCI-EDRN validation cohort, unblinding of the data is not allowed. To maintain patient confidentiality, no individual level data will be provided, only patient summaries.
